# Fullerene Derivatives of Nucleoside HIV Reverse Transcriptase Inhibitors—In Silico Activity Prediction

**DOI:** 10.3390/ijms19103231

**Published:** 2018-10-19

**Authors:** Aleksandra Dąbrowska, Tomasz Pieńko, Przemysław Taciak, Katarzyna Wiktorska, Zdzisław Chilmonczyk, Aleksander P. Mazurek, Adam Stasiulewicz

**Affiliations:** 1 National Medicines Institute, Chełmska 30/34, 00-725 Warsaw, Poland; aleksandra_dabrowska@o2.pl (A.D.); k.wiktorska@nil.gov.pl (K.W.); z.chilmonczyk@nil.gov.pl (Z.C.); apmprofesor@gmail.com (A.P.M.); 2 Medical University of Warsaw, Żwirki i Wigury 61, 02-091 Warsaw, Poland; tom.pienko@gmail.com (T.P.); przemek.taciak@gmail.com (P.T.); 3 Centre of New Technologies, Banacha 2c, 02-097 Warsaw, Poland

**Keywords:** HIV, reverse transcriptase, NRTI, fullerenes, molecular docking, drug design

## Abstract

Here we present new derivatives of nucleoside reverse transcriptase inhibitors with a C_20_ fullerene. The computational chemistry methods used in this study evaluate affinity of designed compounds towards the HIV-1 reverse transcriptase (RT) binding site and select the most active ones. The best of the designed compounds have superior or similar affinity to RT active site in comparison to most active test compounds, including drugs used in anti-HIV therapy.

## 1. Introduction

Human immunodeficiency virus (HIV) is still a major global public health issue. In 2014, 36.9 million people were living with this virus. Every year, 2 million people become infected with HIV and 1.2 million people die from HIV-related causes globally [1].

HIV infects helper T cells, macrophages, and dendritic cells, the major cells of the human immune system [2]. When CD4^+^ T cell numbers declines below a critical level, cell-mediated immunity is lost, and the body becomes progressively more susceptible to opportunistic infections. The late symptoms of the infection are referred to as acquired immunodeficiency syndrome (AIDS) [3].

The global epidemic has prompted virologists to understand the pathogenic processes caused by the virus, determine its structure, as well as search for preventive vaccines and antiviral drugs. Knowledge of the viral enzymes responsible for the replication of the virus (reverse transcriptase, protease, integrase) allows for synthesis and fast introduction of drugs, which are inhibitors of these active proteins, into therapy.

Reverse transcriptase inhibitors prevent the HIV enzyme reverse transcriptase (RT) from converting single-stranded HIV RNA into double-stranded DNA. There are two types of RT inhibitors. Nucleoside/nucleotide RT inhibitors (NRTIs) are faulty DNA building blocks that stop HIV DNA synthesis. Non-nucleoside RT inhibitors (NNRTIs) bind to RT, interfering with its ability to convert HIV RNA into HIV DNA.

Nucleoside reverse transcriptase inhibitors are derivatives of 2′,3′-dideoxynucleosides. They are active only in the triphosphate form [4]. Human kinase-mediated phosphorylation occurs at the 5′ position in three stages [5]. NRTIs are substrates for the enzyme and are incorporated into the DNA chain. Due to the lack of a hydroxyl group at the 3′ position, formation of a phosphodiester bond with another nucleoside is impossible, and further elongation of the nucleic acid cannot occur, thereby inhibiting viral replication. Lamivudine and emtricitabine are structurally remote from 2′-deoxynucleosides. Instead of deoxyribose, they contain oxathiolane, which also prevents further elongation of the DNA.

Fullerene is an allotrope of carbon. The fullerene molecules are composed of even number of carbon atoms that form a spherical polyhedron. C_20_ is the smallest possible fullerene. It is composed of 12 pentagons and has a structure of a regular dodecahedron with a diameter of about 3.1 Å. The C_20_ molecule has 30 C-C bonds of about 1.46 Å. Next to C_60_, C_20_ has the most regular geometry of all fullerenes [6].

The latest research shows that fullerene derivatives could be novel, great antiviral compounds to treat the human immunodeficiency virus infection [7,8]. HIV protease and reverse transcriptase enzymes are the two targets for anti-HIV fullerene derivatives. Molecular modeling studies have demonstrated that C_60_ derivatives could act as HIV RT inhibitors [7,9,10]. In 2005, Mashino et al. [11] synthesized various malonic acid- and amino acid-type C_60_ derivatives and investigated the inhibitory activity of those compounds towards HIV RT. Machino continued his fullerene research, and in 2015, showed that pyridine/pyrimidine-type fullerene derivatives could inhibit the enzyme without cytotoxic effects [12].

The fullerene derivatives should therefore be considered new chemical entities with new biological activity as a result of overall structure rather than composition of two subcomponents. Here we performed in silico molecular modeling studies on compounds formed with C_20_ fullerene and lamivudine, zidovudine, and emtricitabine.

**Figure 1:**
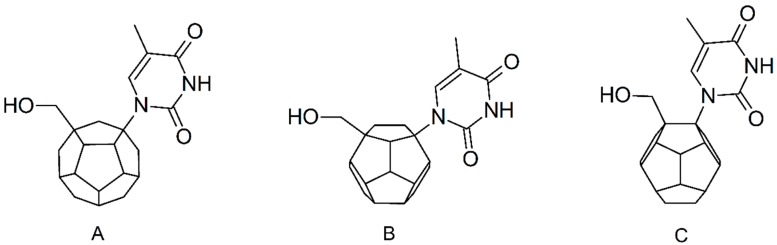
Substitution of C_20_ by purine or pyrimidine bases can occur in three ways. In order to increase the readability, hydrogen atoms and double bonds in the fullerene part were left out. (**A**) Substitution based on the structure of most nucleoside/nucleotide RT inhibitors (NRTIs), such as zidovudine. (**B**) Substitution based on the structures of lamivudine and emtricitabine. (**C**) The third type of substitution.

Studied ligands were designed on the grounds of nucleoside reverse transcriptase inhibitors’ structures. The key difference in our studies is the replacement of dideoxyribose ring or oxathiolane with a C_20_ fullerene. Three leading structures are proposed. They are built by pyrimidine or purine base and hydroxymethyl group attached in various positions to one side of C_20_. This is illustrated in Figure 1. The first type of substitution is based on the structure of most NRTIs, such as zidovudine. The second type is based on the structures of lamivudine and emtricitabine. Additionally, there is proposed also a third type of connection. Three bases were used: adenine, thymine, and cytosine. Additionally, non-substituted carbon atoms of the substituted fullerene rings were replaced with atoms of nitrogen, sulfur or boron. We also propose compounds with a methylene group connecting fullerene with the nitrogenous base. All the combinations used give 180 compounds in total. Replacement of one carbon atom in C_20_ with a nitrogen atom causes a loss of a double bond and addition of one hydrogen atom. In order to increase the comparability of the results, sulfur and boron derivatives as well as derivatives without heteroatoms have one double bond saturated, adding two hydrogen atoms. As a result, the fullerene core in every designed compound has exactly 8 double bonds.

In order to evaluate the binding affinities of designed compounds towards RT active site, an in silico study was performed. The fullerene derivatives were docked to multiple reverse transcriptase models. Selected molecules had their binding free energies calculated. Then, the best protein-ligand complexes were analyzed, giving valuable insight into interactions between studied compounds and RT active site.

## 2. Results and Discussion

**Figure 2:**
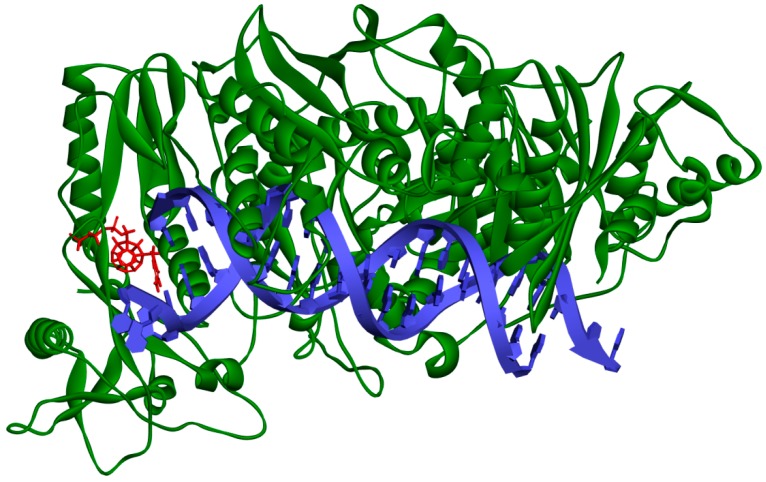
Studied compound (red) docked to the HIV-1 reverse transcriptase (green). Blue represents DNA.

**Figure 3:**
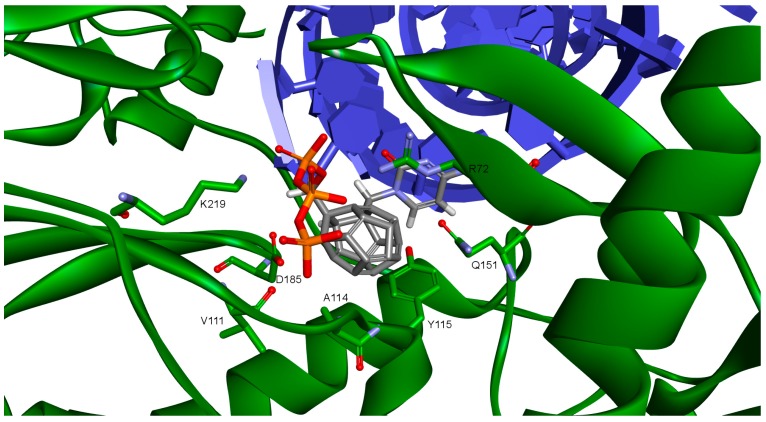
Studied compound docked to reverse transcriptase. View of the active site. Green represents reverse transcriptase (RT), blue DNA. Studied fullerene derivative along with the most important amino acids located at the binding site are shown in stick representation.

One hundred eighty designed compounds were docked to four RT models. Docking protocol used in this study includes ligand flexibility, yet the protein remains rigid. In order to imitate flexibility of amino acids of the active site, rigid docking was performed for different RT conformations. This procedure minimized the occurrence of false negative results. It is worth mentioning that among these models are two generated by us. During the validation of the docking protocol, four new RT models were prepared. For best of them (Model 3) the Pearson correlation coefficient between Ki values and scoring function values of test compounds proved to be superior than for the crystallographic structures. In Tables S13–S16 all docked fullerene derivatives are presented along with potential of mean force (PMF) and PLP1 function values. Figure 2 and Figure 3 show how studied compounds are placed in RT.

**Figure 4:**
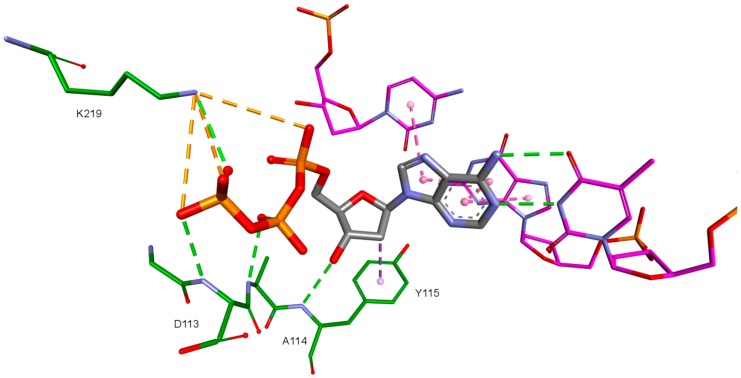
dATP orientation in the RT active site. 3KK2 crystallographic structure. RT amino acids: green; DNA nucleotides: purple.

**Figure 5:**
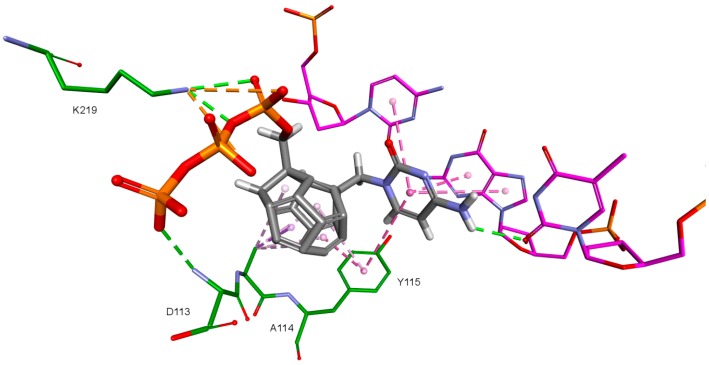
Orientation of compound 5 docked to model based on 3KK2 crystallographic structure. RT amino acids: green; DNA nucleotides: purple.

Ligands that achieved best scores had their orientations in the binding pocket evaluated and then those with orientations similar to orientations of ligands in the crystallographic structures were chosen. Orientations of NRTI and well-placed ligand are shown in Figure 4 and Figure 5. The necessity of the above action results from the mechanism of action of NRTIs. They are built into the DNA strand, which requires not only strong affinity towards binding sites, but also adequate orientation allowing formation of a phosphodiester bond between ligand molecule and DNA strand nucleotide. After considering both factors, these are the compounds most promising to be active towards RT (The ordinal numbering from Tables S13–S16).

1. **3KK2**: 6, 78, 2, 25, 102, 106, 174, 28, 7, 107, 83, 48
2. **Model 2**: 29, 47, 5, 83, 66, 78, 82, 30, 4, 94, 144, 100
3. **Model 3**: 6, 78, 102, 84, 60, 114, 72, 107, 76, 96, 105, 83
4. **3V4I**: 18, 3, 27, 25, 132, 40, 39.

**Figure 6:**
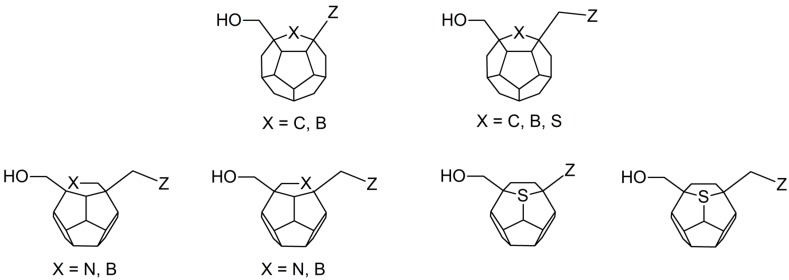
Structures of the best ligands. “Z” stands for nitrogenous base: thymine, cytosine or adenine. In order to increase the readability, hydrogen atoms and double bonds in the fullerene part were left out.

PMF scoring function values for 3KK2, Model 2 and Model 3 indicate that the best designed ligands have a higher affinity towards RT than test compounds, including zidovudine and stavudine triphosphates. On the other hand, the PLP1 function values for 3V4I model show that best designed compounds have similar affinity towards RT compared to the most active test ligands. Among the above-mentioned compounds a few derivative groups occur most frequently. It allows for the selection of favorable structural features. These are mainly the derivatives with the hydroxymethyl groups and nitrogenous bases attached to the fullerene core in positions analogical to NRTI structures. Compounds designed that are not based on existing NRTI structures, proved to have weaker affinity towards the RT active site. Presence of the heteroatom in the fullerene core turned out to be another important feature. Depending on the substitution position of the two aforementioned groups to the fullerene, different heteroatoms and different places of their insertion are preferred. In many cases the addition of the methylene group between the fullerene core and the nitrogenous base proved to be beneficial. Groups of the best ligands are shown in Figure 6.

**Figure 7:**
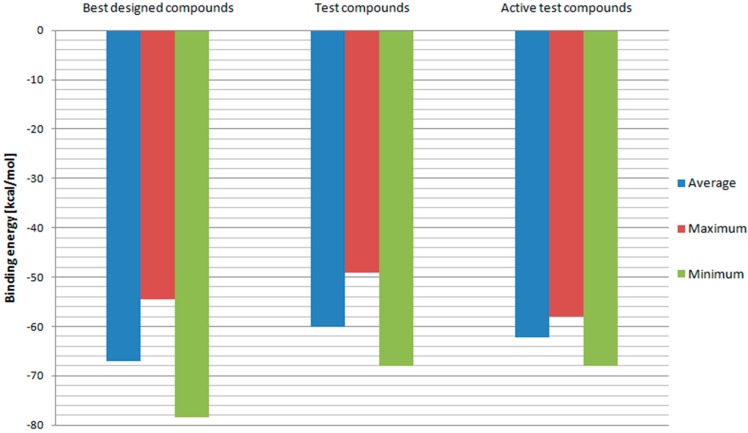
Binding free energies calculated for Model 3. “Best designed compounds” include top scored fullerene derivatives from docking. “Active test compounds” are test compounds with Ki less than 100 nM.

In order to further evaluate and confirm the docking results, binding free energies of above-mentioned compounds and test ligands were calculated. The RT model with the best correlation for Ki values and docking scores—Model 3 was used in this part of the research. The results are depicted in Figure 7. Designed ligands with best docking scores proved to have on average more negative binding energy values when compared to test ligands. While the test ligands’ set contains also those with low binding affinities, binding energies of designed ligands were additionally compared with those of test ligands with Ki values below 100 nM. Once again, fullerene derivatives managed to receive on average superior results. Also, minimum binding energy for designed compounds turned out to be more negative than the test one. This means that fullerene derivatives should have a higher affinity towards HIV RT. However, the maximum value so far of the best designed compounds was less negative than some of the test ligands’ binding energies. Therefore, the set of potential RT inhibitors was further reduced to include compounds: 2, 3, 4, 5, 6, 18, 29, 47, 72, 76, 78, 82, 83, 84, 94, 96, 100, 102, 106, 114, and 144. Their structural formulas along with scoring function values and binding energies are shown in Table 1.

Molecular docking allows also to analyze the interactions between ligands and amino acids in binding sites. Docking of the ligand base to four RT models allows the enhanced examination of these interactions, because they can differ with respective proteins conformations. In the case of RT active site, ligands interact also with DNA nucleotides. Most of the interactions are common for all four used RT conformations, therefore they were investigated together for all RT models. In the case of the occurrence of significant differences, they were specified and explained.

**Figure 8:**
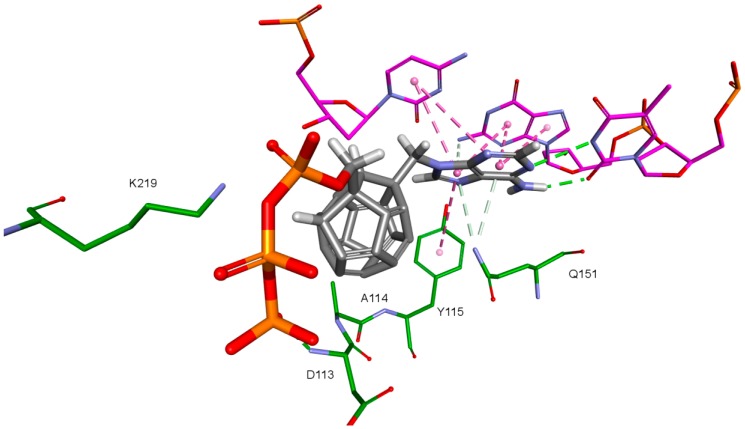
Interactions of the nitrogenous base. Model 3, compound 6. RT amino acids: green; DNA nucleotides: purple.

Nitrogenous bases in the studied compounds interact primarily with both strands of DNA nitrogenous bases. It is worth mentioning that scoring function values and orientations of the ligands were not strongly influenced by the type of the substituted nitrogenous base. Ligands were placed in the binding pocket well, regardless of whether the used nitrogenous base was complementary to the base in the template DNA strand or not. It could be the result of the presence of the fullerene core. It makes a significant difference in comparison to deoxyribose rings of the RT natural ligands. It certainly affects the geometry of the compound as well as its orientation in the active site, which may improve the nitrogenous base’s ability to obtain favorable position, regardless of its complementarity. For derivatives with a methylene group between the nitrogenous base and the fullerene core, this modification can also alter the orientation of the nitrogenous base. There are hydrophobic π-π stacking interactions between base rings of the ligands and nucleotide base rings of DNA primer strands. Hydrogen bonds occur between ligands’ bases and bases to be complementary. There are also π–π stacking interactions between base rings of ligands and nucleotide base rings of the template strand, that are situated near the nucleotides supposed to be complementary. Additionally, some ligands tend to create π–π T-shaped interactions. They are formed by π electrons of the base ring of the ligand and tyrosine 115 ring, that are oriented perpendicularly to each other. In some cases, hydrogen bonds between glutamine 151 hydrogen atom and π electrons of the nitrogenous bases rings can be observed. The last occurring interaction type is an electrostatic cation–π interaction, formed by electrons of nitrogenous bases and protonated imine group =NH_2_^+^ of arginine 72. The interactions of the nitrogenous bases are illustrated in Figure 8.

**Figure 9:**
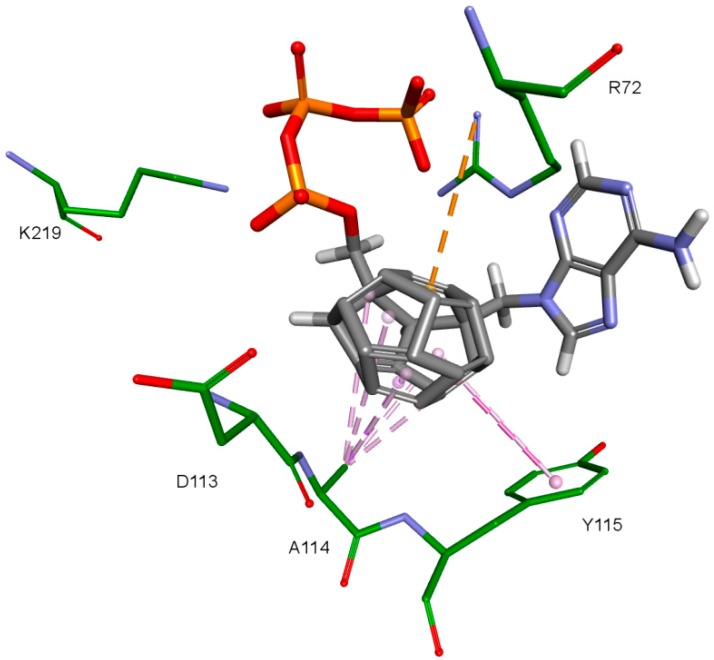
Interactions of the fullerene core. 3KK2, compound 6. RT amino acids: green.

The key difference between the designed compounds and the currently used NRTIs is the replacement of the dideoxyribose ring with a fullerene. For this part of the compound, the biggest differences in interaction with amino acids at binding site occur when compared to nucleosides or NRTIs. Interaction of hydrophobic π electrons of fullerene cores with alanine 114 methyl group is most frequently observed. It occurs in all ligand-protein complexes, for all RT models. The interaction between fullerene core and tyrosine 115 ring has to be mentioned as well, as it belongs to the most significant ones. It is of hydrophobic nature π–π stacking group and occurs for all of the prepared RT models. However, in the case of Model 3 it is observed rarely, only for a limited portion of the studied ligands. This suggests the dependence of the occurrence of the aforementioned interaction on amino acid side chains’ conformations in the β3-β4 flexible loop in p66 fingers subdomain. Also, for some ligands hydrogen bonds occur between the fullerene part and a hydrogen atom in the peptide bond between alanine 114 and tyrosine 115. The above-mentioned interactions of the fullerene core are only the main ones. In the case of certain orientations of specific ligands interactions also occur with other amino acids in the binding pocket. Relatively often electrostatic cation-π interactions with lysine 65 amine group can be observed. They have partially hydrogen bond character. There are also cation-π interactions with =NH_2_^+^ group and hydrogen bonds with amine group of arginine 72. Additionally, hydrophobic interactions with two aforementioned amino acid side chains occur. Another type of fullerene core’s interaction is that of its π electrons with free electron pairs of glutamine 151 oxygen atom as well as with oxygen atom in the peptide bond between valine 111 and glycine 112. The other observed interaction is the one between the fullerene core and aspartic acid 185 which belongs to the anion-π type. Interactions of the fullerene core are depicted in Figure 9.

**Figure 10:**
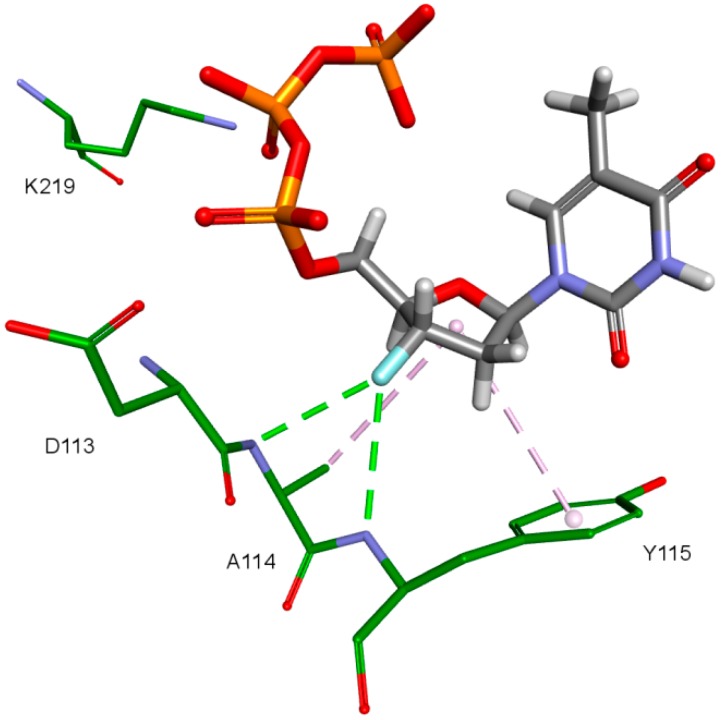
NRTI interactions. 3KK2, test compound 3-F-dTTP. RT amino acids: green.

The fullerene core’s numerous interactions with the active sites amino acids are probably the main cause of positive results in form of scoring function values. Natural RT ligands and NRTIs form usually only two to three interactions with their deoxyribose or dideoxyribose ring and function groups attached in its position 3. They are shown in Figure 10. These are mainly hydrophobic interactions of the carbohydrate ring with tyrosine 115 ring as well as hydrogen bonds and electrostatic interactions of the hydroxyl or azide groups. It is definitely less than in the fullerene part of the designed compounds.

**Figure 11:**
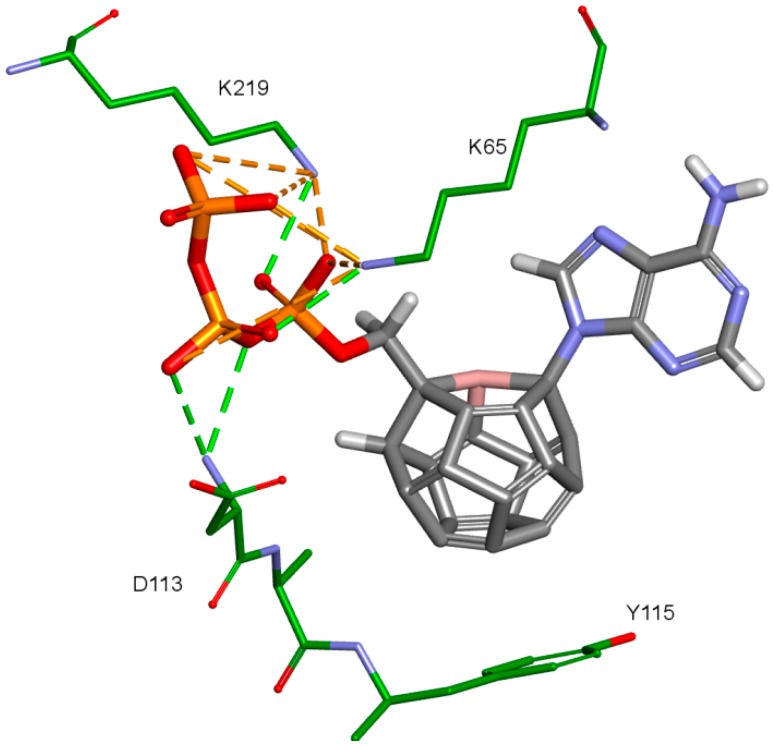
Interactions of the triphosphate group. 3V4I, compound 27. RT amino acids: green.

The third important group of interactions in the studied compounds that occurs in ligand–protein–DNA complexes includes the triphosphate group interactions. This part of the molecule was not modified. Its interactions do not differ from those commonly observed in NRTIs and RT natural ligands. They depend mostly on orientation of the triphosphate part in the active site. These are primarily hydrogen bonds and electrostatic interactions. All of the oxygen atoms in the triphosphate group can take part in the formation of these interactions. The hydrogen bonds’ donor atoms are located in the peptide bonds between glycine 112 and aspartic acid 113, lysine 65 and lysine 66 as well as lysine 66 and aspartic acid 67. Additionally, bonds of this type can be formed due to the hydrogen atoms present in glycine 112, in the side chains of lysine 219 and lysine 66, and in arginine 72 amine group. Hydrogen bonds also are present, formed by the electrostatic interactions between oxygen atoms of the triphosphate group and lysine 65 and lysine 219 amine group and arginine 72 =NH_2_^+^ group. It is worth mentioning that the interactions of the triphosphate group depend not only on its orientation in the active site, but also on the conformations of the amino acid side chains in the binding pocket. For example, in the case of Model 3, in which Lys65 and Arg72 conformations were altered, there are no interactions of the triphosphate group with arginine 72. This is caused by translation of this amino acid side chain in the direction of the DNA template strand and increasing the distance from amino acids important for triphosphates binding, such as Lys219. This change prevents the interaction of Arg72 with part of the ligand, regardless of its orientation in the binding pocket. In the Model 3V4I lysine 66 and aspartic acid 67 are placed far from the binding site and do not contribute to the creation of the ligand-protein complex at all. Interactions of the triphosphate group are depicted in Figure 11.

**Figure 12:**
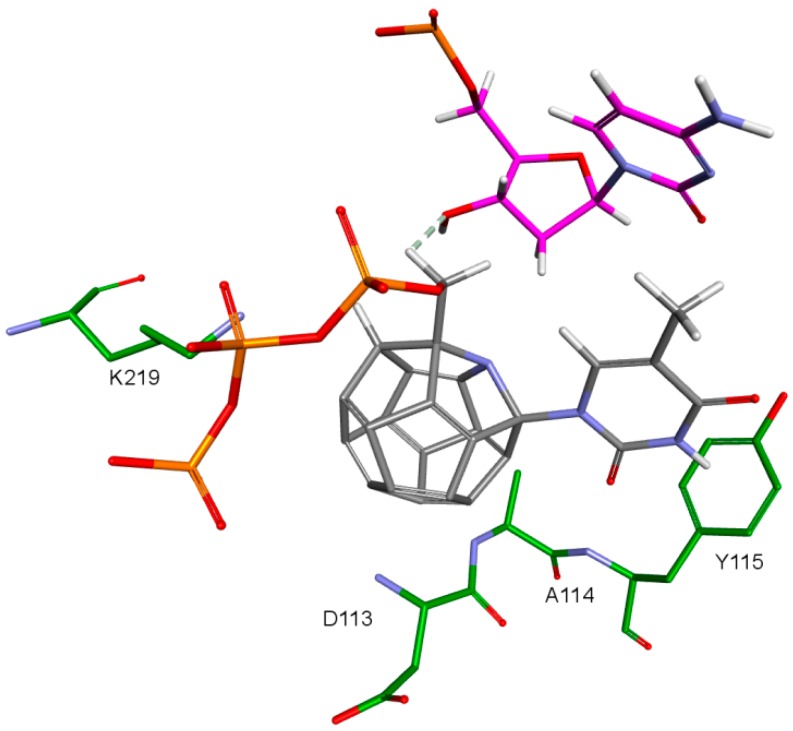
Hydrogen bond between ligand’s methyl group and deoxyribose’s hydroxyl group. 3KK2, compound 7. RT amino acids: green; DNA nucleotides: purple.

An important factor for achieving by the designed compounds the correct orientation in the RT binding site is the methylene group connecting the fullerene part with the triphosphate group. The hydrogen atom forms a hydrogen bond with the oxygen atom in the deoxyribose of DNA primer strand to which the ligand is supposed to be attached. This is illustrated in Figure 12. Hydrogen atoms of the methylene group sometimes form also the hydrogen bond with oxygen atom of the carboxyl group of aspartic acid 185.

## 3. Materials and Methods

### 3.1. Ligands’ Preparation

Due to the NRTIs mechanism of action, all designed compounds were prepared for docking in the form of triphosphates. All designed compounds’ structures are shown in Table S1 as Supplementary Materials. The structures were created in Discovery Studio 4.1 program. Their geometry was optimized in a default force field of this program, similar to DREIDING [13] force field and triphosphate groups were adequately ionized.

### 3.2. RT Models’ Preparation

Crystallographic structures of HIV-1 reverse transcriptase were obtained from Protein Data Bank (www.rcsb.org) [14]. 6 structures of RT-DNA complexes were chosen: 3V4I [15], 1RTD [16], 1T05 [17], 3KK1, 3KK2 and 3KJV [18]. All of them, except 3KJV, contain also ligands in the active sites of the enzymes. In order to determine 3D structure of the complex, primer DNA strands in these complexes are ended with dideoxynucleosides or incorporated NRTIs. Table 2 contains more information on chosen RT structures.

Structures of protein complexes were imported to Discovery Studio 4.1. The first part of model preparation was the exchange of terminal nucleotides of primer DNA strands for normal nucleotides with hydroxyl group in position 3′. Protein structures were then optimized with Prepare Protein protocol. This operation included deletion of ligands, water and postcrystallization artifacts, optimization of loops, adding hydrogens and ionization of amino acid side chains. For these purposes CHARMm (Chemistry at Harvard Macromolecular Mechanics) [19] force field was used. Next step included determination of the binding site. It was defined as a sphere with 10 Å radius with the middle in the geometrical center of selected elements: Lys65, Arg72, Asp110, Val111, Gly112, Asp113, Ala114, Tyr115, Gln151, Asp185, Lys219 and 2 nucleotides in DNA template strand near Tyr115 and Gln151.

### 3.3. Model Validation and Selection of Scoring Functions

In order to validate prepared protein models, docking of ligands with known affinity towards RT was performed. Twenty test ligands with known Ki values were chosen [20]. They are illustrated in Table S2 in Supplementary Materials. Structures of these compounds were downloaded from PubChem [21] base and later imported to Discovery Studio 4.1. Docking was performed with CDOCKER [22] protocol. Ligand-protein-DNA complexes were scored with seven functions: LigScore1, LigScore2, PLP1, PLP2, Jain, PMF and PMF04. One best scored complex was chosen for each ligand. Scoring function values were correlated with Ki inhibition constants. Pearson correlation coefficients were then calculated. More information on scoring functions results for test compounds contain Tables S3–S8. Best Pearson correlation coefficient was achieved for 3KK2 model and PMF function. It was −0.57143. Value below −0.5 was achieved also for 3V4I model with PLP1 function. The rest of the best values for the rest of the models fall between −0.4 and −0.5.

### 3.4. Preparation of New RT Models

Based on the RT model with best Pearson correlation coefficient for Ki values and scoring function values, which was 3KK2, new RT models were prepared. There were created four new models with altered conformations. Two of them were conceived with Side Chain Refinement protocol. The other two models were prepared via manual change of selected amino acids side chains conformations, using Ponder and Richards [23] rotamer library. Table 3 contains more details on these four new models. They were tested analogically to previous RT models. Information on scoring function values for test compounds and new RT models is in Tables S9–S12 in the Supplementary Materials. Special attention was deserved by Model 3, which used with PMF scoring function achieved −0.66910 Pearson correlation coefficient, which is a better value than this of 3KK2. The value below −0.5 was also achieved for Model 2 and PMF function.

### 3.5. Docking

Docking of designed ligands was performed with Discovery Studio 4.1, using CDOCKER protocol. The following HIV RT models were used: 3KK2, 3V4I, and Model 2 and Model 3 based on 3KK2 structure. In order to evaluate ligand-protein complexes, adequate scoring functions were used. These were the ones with best Pearson correlation coefficients for Ki values and scores for test compounds. In case of 3KK2 and Models 2 and 3 it was PMF, in case of 3V4I-PLP1. Ligands with best scores for each RT model had their orientations in the binding pocket evaluated. Those with orientations similar to orientations of ligands in the crystallographic structures were chosen.

### 3.6. Binding Free Energy Calculations

Poses with best PMF scores, obtained after docking to Model 3, were chosen for binding energy calculations. The ligands were first minimized in the presence of HIV RT. It was performed with Discovery Studio 2018, using In Situ Ligand Minimization protocol. Forcefield was specified as CHARMm and protein’s hydrogens were set as flexible. Then, binding energy calculations were carried out with the same program, using Calculate Binding Energies protocol. Generalized Born with Simple SWitching (GBSW) implicit solvent model was chosen. For comparison, binding free energies of test ligands were also evaluated.

## 4. Conclusions

We designed 180 new derivatives of nucleoside reverse transcriptase inhibitors with a C_20_ fullerene. Computational chemistry methods used in this study allowed us to evaluate the possible affinity of designed compounds towards HIV-1 RT binding site and select the most active ones (Table S1: 4, 6, 29, 47, 72, 76, 82, 84, 96, 10, 106, 114). One hundred eighty designed compounds were docked to four chosen RT models. The best of the designed compounds had potentially superior or similar affinity to RT active site in comparison to most active test compounds, including drugs used in anti-HIV therapy. We plan to synthesize selected C_20_ derivatives and examine them in vitro.

This is the first study that indicates potential affinity of fullerene derivatives towards HIV RT active site. While the methods used in this work allow only for preliminary conclusions, it may be an important groundwork for both more accurate studies of the studied compounds and other related research in that area. This is also a pioneering attempt to show a potential application of C_20_ as an active pharmaceutical ingredient. Presented results can serve as starting points for further studies on other chemical groups of compounds containing fullerenes. We intend to extend our research and study other fullerene derivatives, paying special attention to their potential affinity towards HIV-1 RT.

## Figures and Tables

**Table 1:** Designed compounds with best binding energy values. Columns 3–6 show scoring function (lower part of the title row) values for respective RT models (upper part of the title row). Last column shows binding energy values calculated for Model 3. ID numbers match those in Supplementary Materials. In order to increase the readability, hydrogen atoms and double bonds in the fullerene part of the structures were left out.

ID	Structure	3KK2 −PMF	Model 2 −PMF	Model 3 −PMF	3V4I −PLP1	Binding Energy [kcal/mol]
2	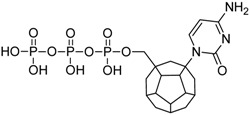	194.64	163.72	162.48	117.20	−65.91
3	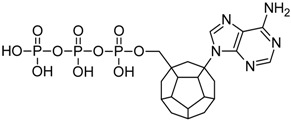	165.14	179.73	165.96	130.55	−69.63
4	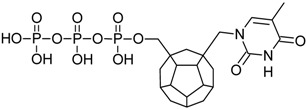	189.20	191.35	169.64	115.46	−71.25
5	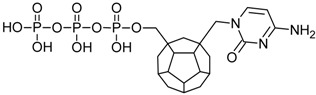	185.71	199.49	164.67	116.01	−67.27
6	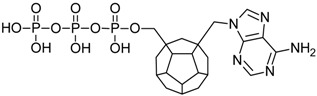	197.88	173.01	185.19	114.23	−70.24
18	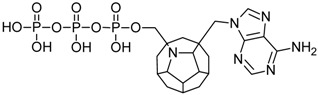	174.92	163.39	149.00	133.72	−66.40
29	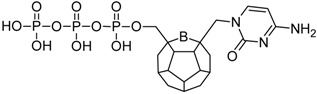	181.57	208.45	166.09	113.58	−72.40
47	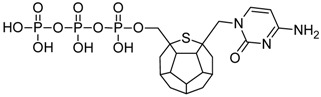	186.98	203.10	162.20	92.20	−72.27
72	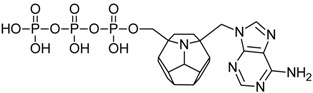	168.74	187.06	174.43	120.65	−76.76
76	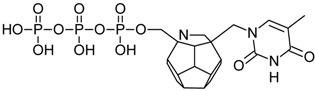	162.71	185.88	173.42	86.63	−71.85
78	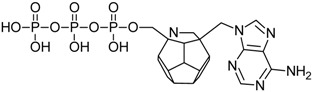	197.16	193.67	183.40	95.51	−68.37
82	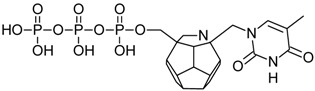	185.56	192.94	167.86	133.56	−73.34
83	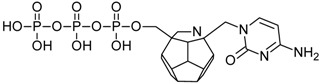	189.50	198.07	172.67	78.95	−65.76
84	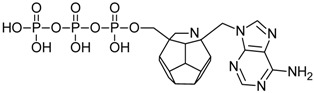	175.09	164.56	177.27	103.63	−70.90
94	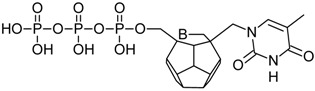	184.85	191.20	168.90	97.54	−66.48
96	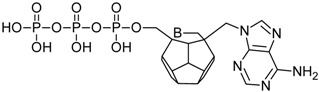	167.58	205.23	173.28	104.58	−73.08
100	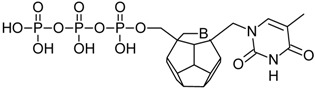	183.42	190.17	166.22	106.16	−68.28
102	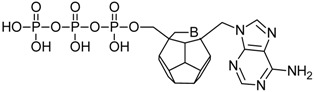	190.89	188.32	179.27	114.29	−78.33
106	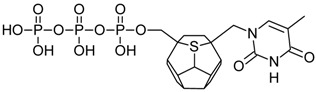	190.73	174.12	168.01	101.99	−72.28
114	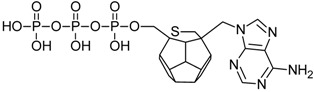	171.32	186.77	175.31	127.41	−74.59
144	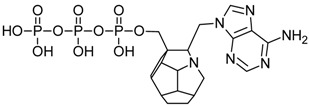	181.78	190.96	158.44	106.51	−67.94

**Table 2:** Reverse transcriptase models.

PDB ID	Ligand	Resolution [Å]
1RTD	dTTP	3.2
1T05	Tenofovir diphosphate	3.0
3KJV	-	3.1
3KK1	GS-9148 diphosphate	2.7
3KK2	dATP	2.9
3V4I	AZTTP	2.8

dTTP—2′-deoxythymidine triphosphate, dATP—2′-deoxyadenosine triphosphate, AZTTP—zidovudine triphosphate, GS-9148-(5-(6-aminopurin-9-yl)-4-fluoro-2,5-dihydrofuran-2-yloxymethyl) phosphoric acid.

**Table 3:** Χ angles of the amino acids with altered conformations in the new RT models.

Amino Acid	3KK2	Model 1	Model 2	Model 3	Model 4
Lys65	−51.24	−162.38	-	−68.90 (40.90%)	-
Lys66	177.09	−44.71	-	-	-
Asp67	89.42	48.67	-	-	-
Arg72	178.95	−169.77	-	−67.60 (46.30%)	-
Asp110	−175.07	−172.63	−171.83	-	-
Val111	167.86	179.67	179.75	-	-
Asp113	−81.13	61.08	−57.58	-	-
Gln151	−64.55	−59.13	-	-	−174.60 (21.10%)
Asp185	75.22	62.93	61.09	-	−68.30 (47.70%)
Lys219	−141.50	−117.40	−118.25	-	−68.90 (40.90%)

Models 1 and 2 were prepared using Side Chain Refinement protocol, Models 3 and 4 using Ponder and Richards rotamer library. “-” means that the particular amino acid remained unmodified. Numbers in brackets depict the probability of the occurrence of each rotamer in Ponder and Richards library.

## Supplementary Materials

Supplementary materials can be found at http://www.mdpi.com/1422-0067/19/10/3231/s1.

## Abbreviations

HIV	Human immunodeficiency virus
AIDS	Acquired Immunodeficiency Syndrome
RT	Reverse transcriptase
NRTI	Nucleoside/nucleotide RT inhibitor
NNRTI	Non-nucleoside RT inhibitor
