# Supplementary material for: Fullerene Derivatives of Nucleoside HIV Reverse Transcriptase Inhibitors—In Silico Activity Prediction
Source: Int J Mol Sci. 2018 Oct 19;19(10):3231. doi: 10.3390/ijms19103231 (PMC6214040; doi:10.3390/ijms19103231)
Supplement: Supplementary file 1 [file ijms-19-03231-s001.pdf]

Table S1: Designed compounds in the form of triphosphates. In order to increase the readibility, hydrogen atoms and double bonds in the fullerene part were left out.

ID	Structure
1	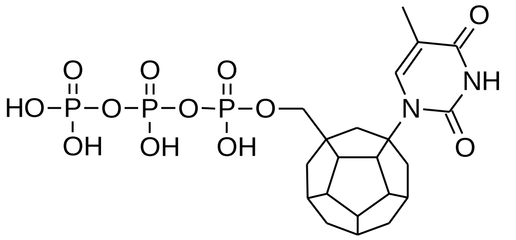
2	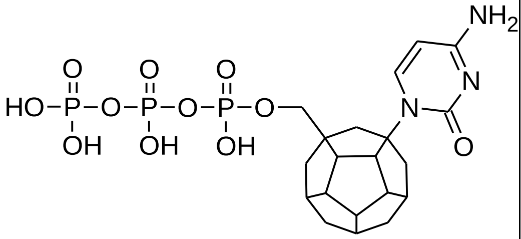
3	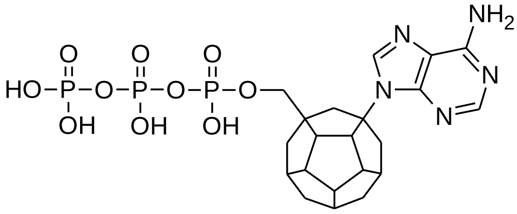
4	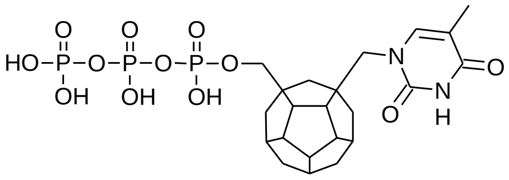
5	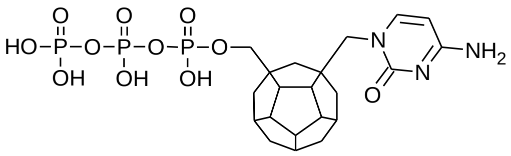
6	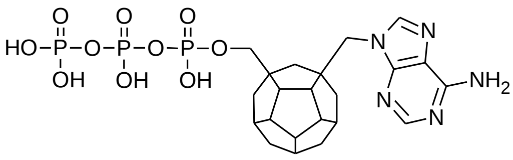
7	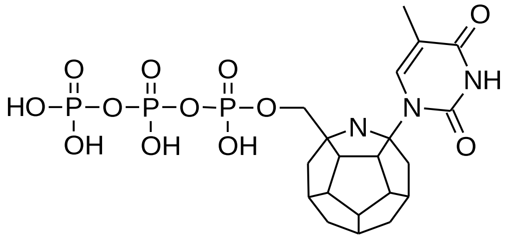
8	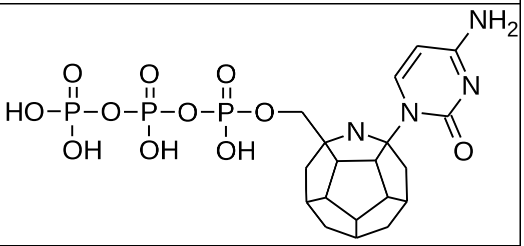
9	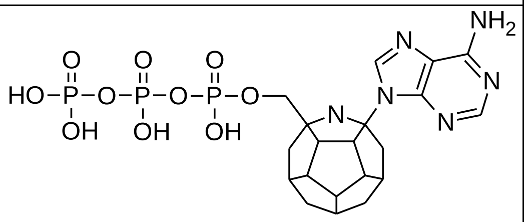
10	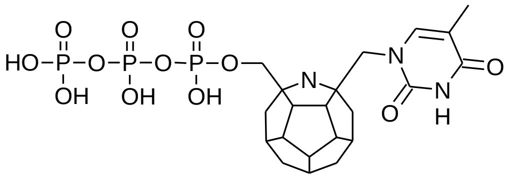
11	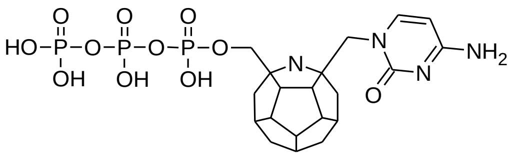
12	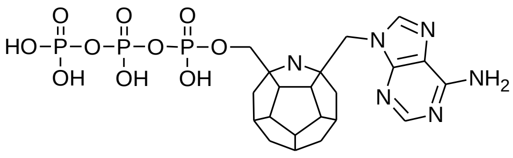
13	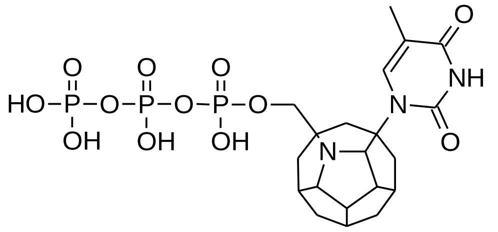

14	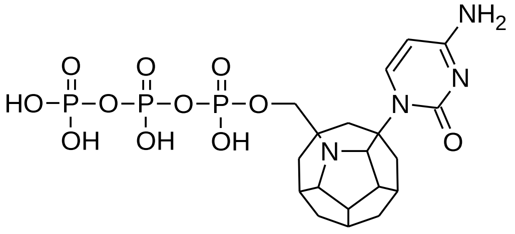
15	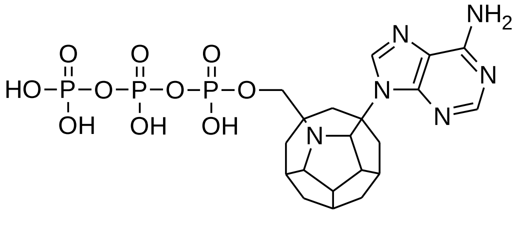
16	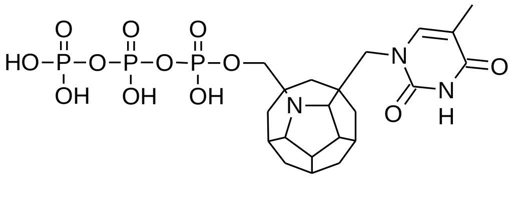
17	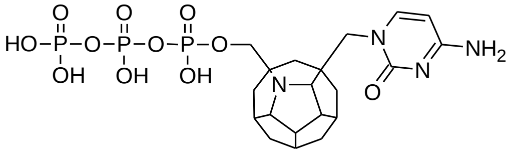
18	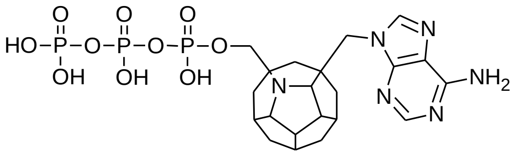
19	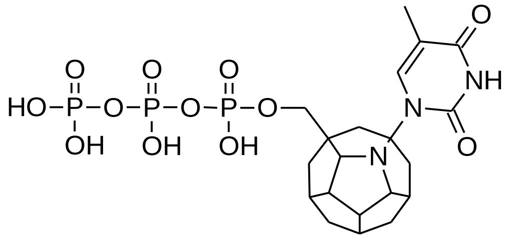
20	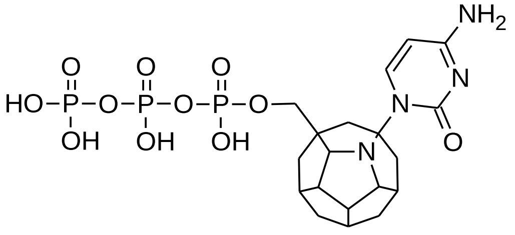
21	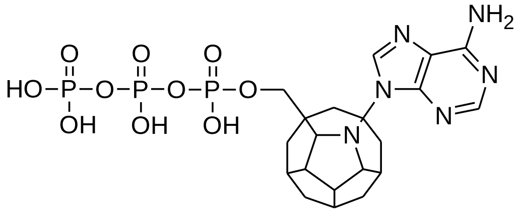
22	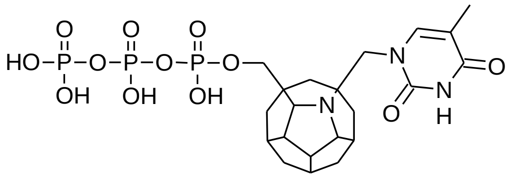
23	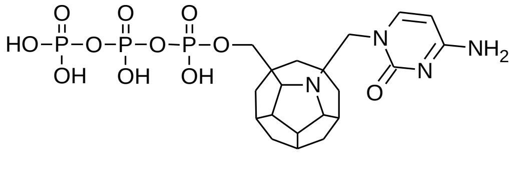
24	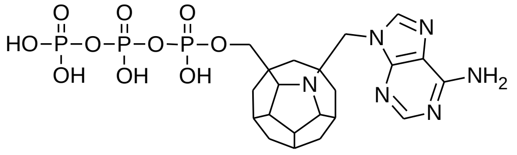
25	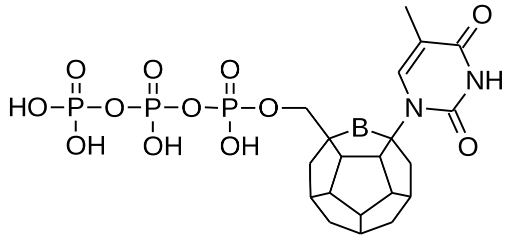
26	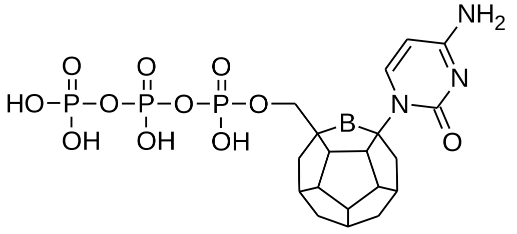
27	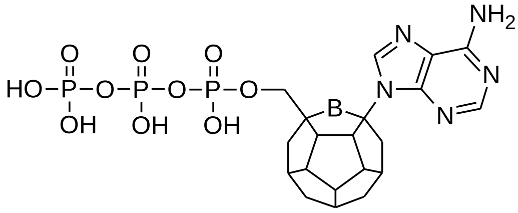

28	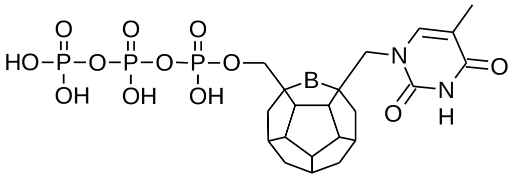
29	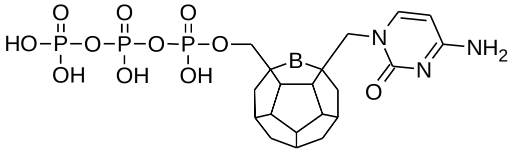
30	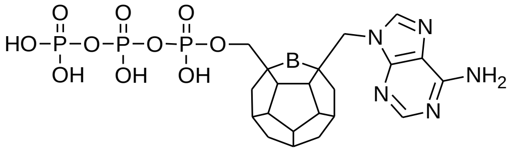
31	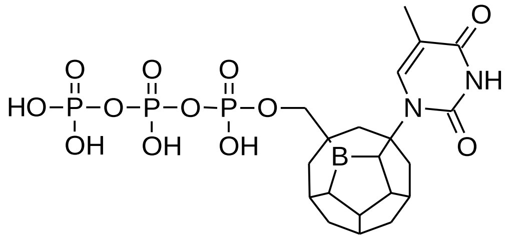
32	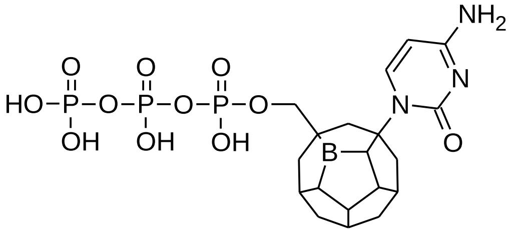
33	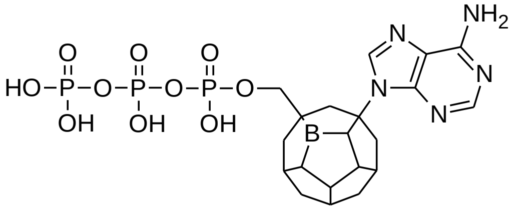
34	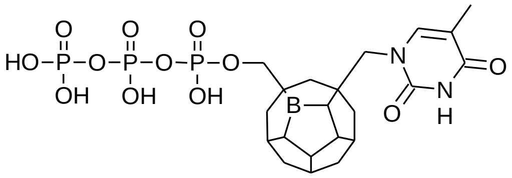

35	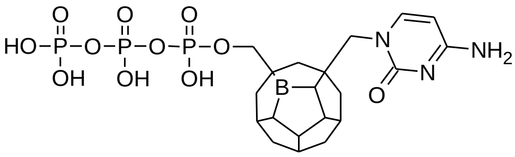
36	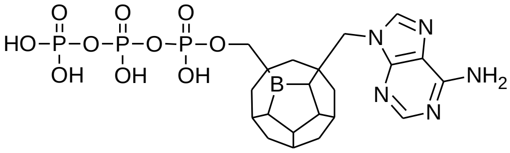
37	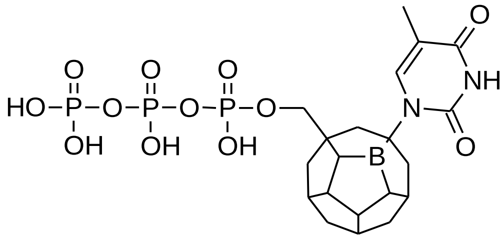
38	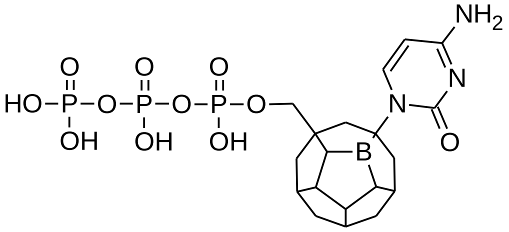
39	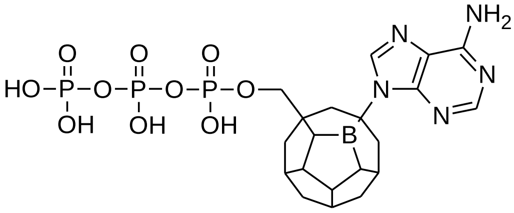
40	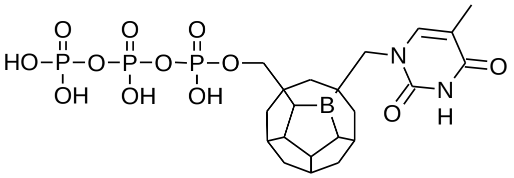
41	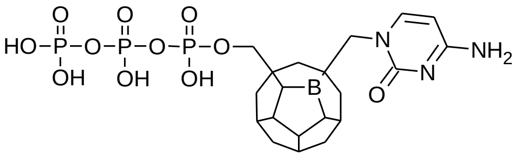

42	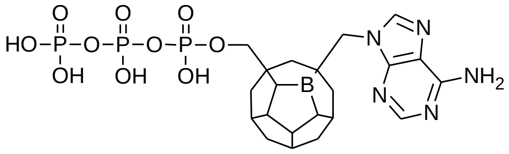
43	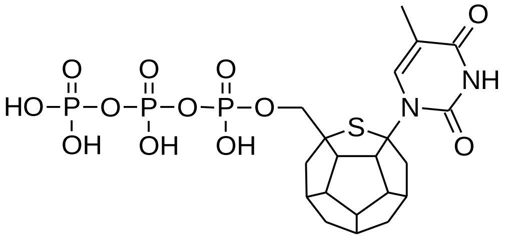
44	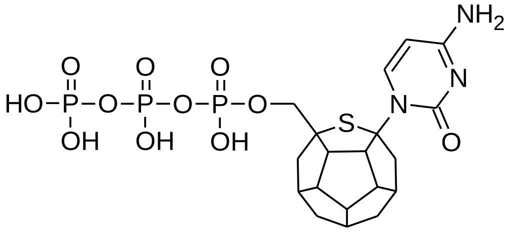
45	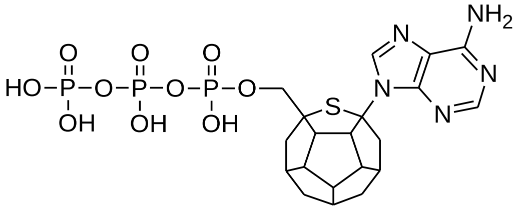
46	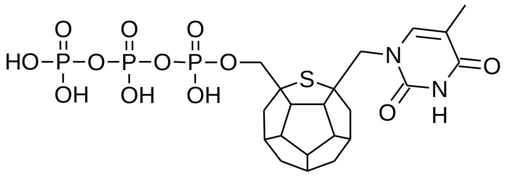
47	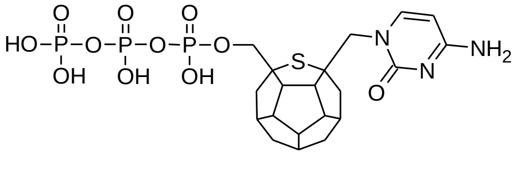
48	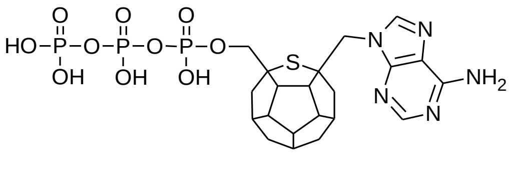

49	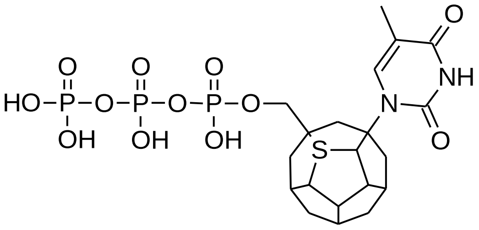
50	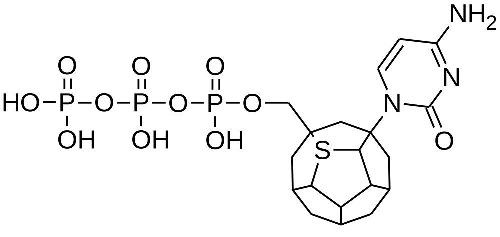
51	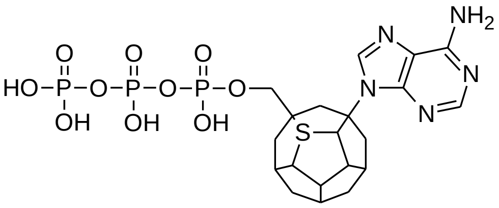
52	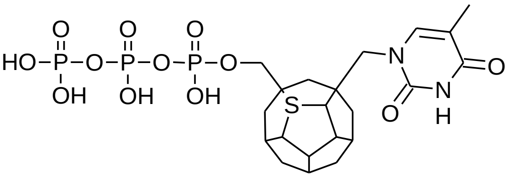
53	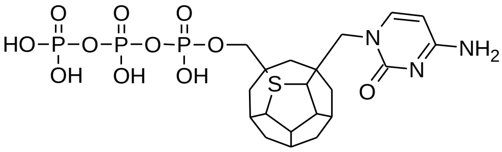
54	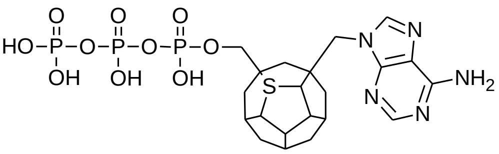
55	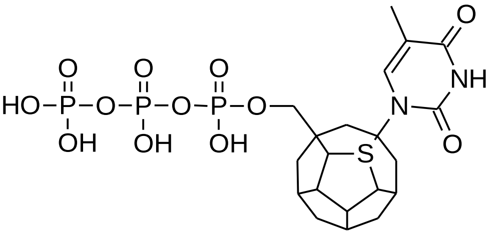

56	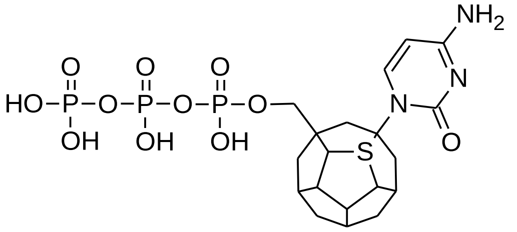
57	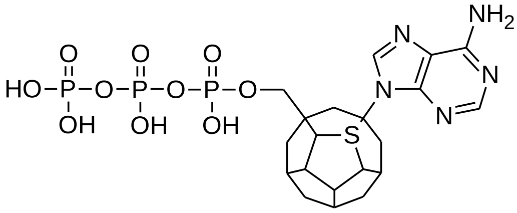
58	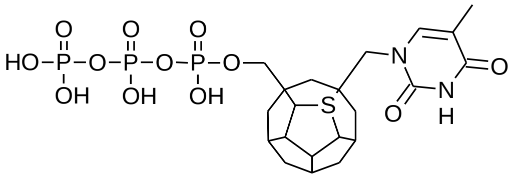
59	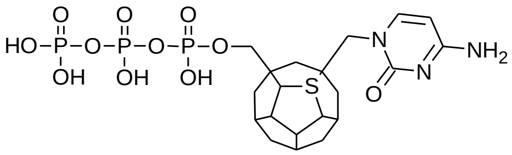
60	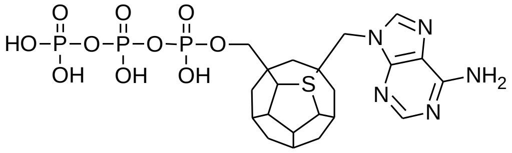
61	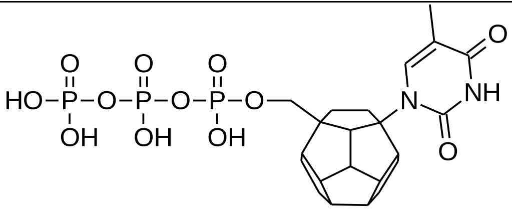
62	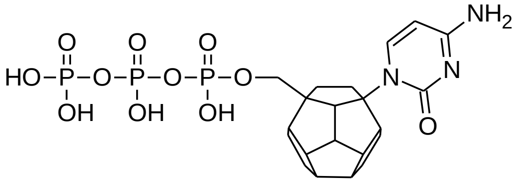
63	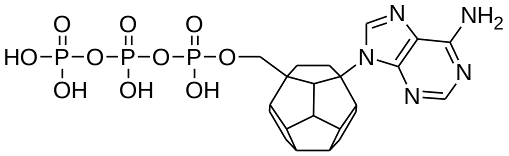
64	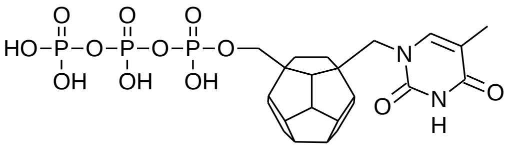
65	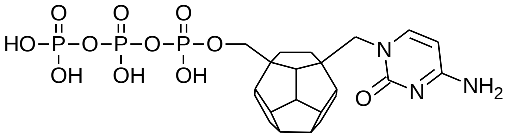
66	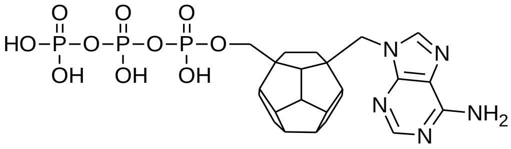
67	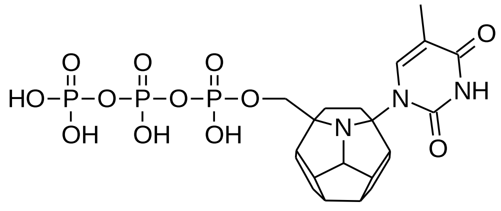
68	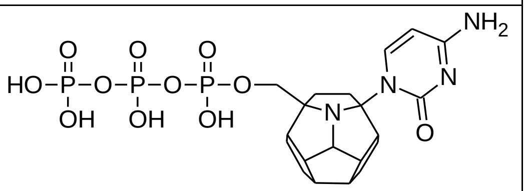
69	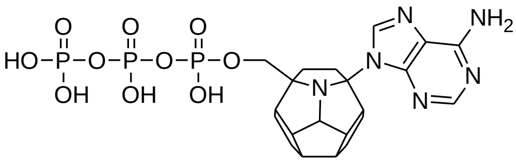

70	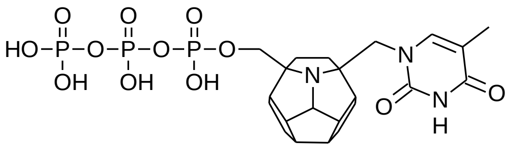
71	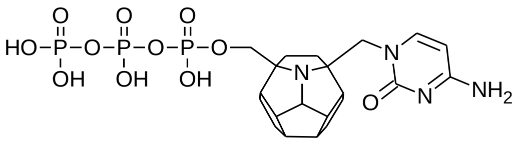
72	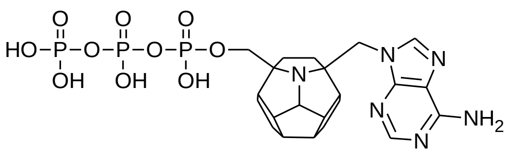
73	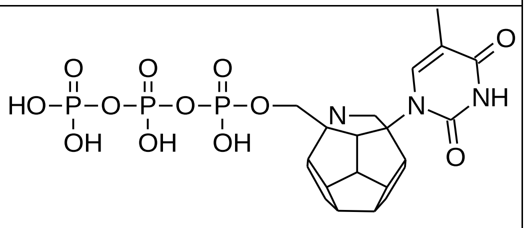
74	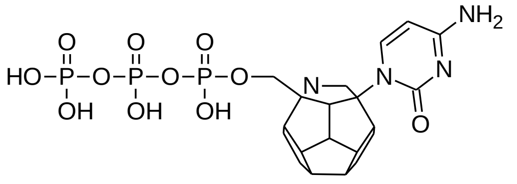
75	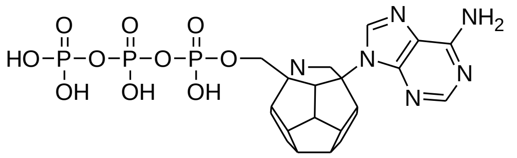
76	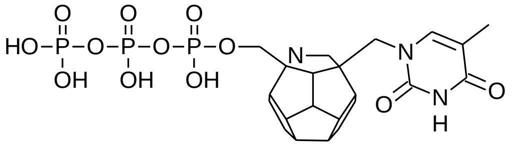
77	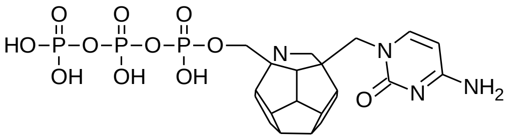
78	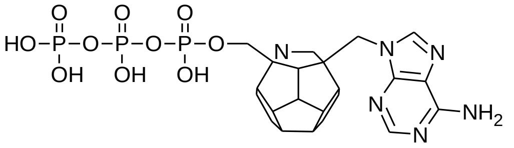
79	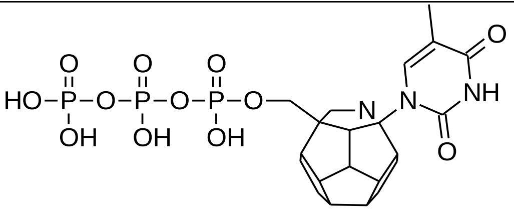
80	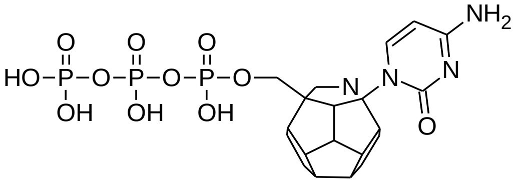
81	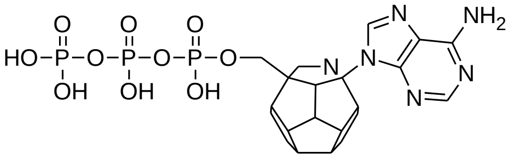
82	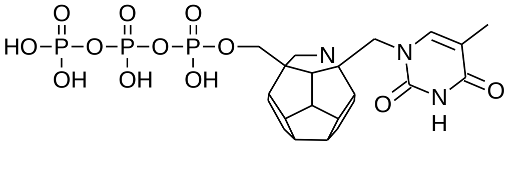
83	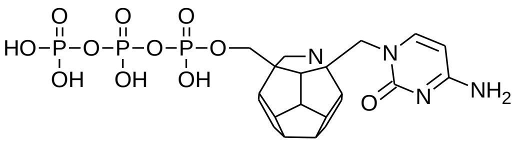

84	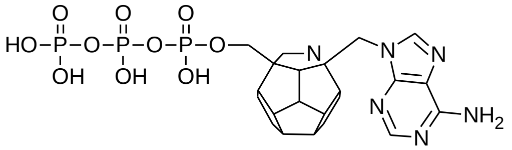	91	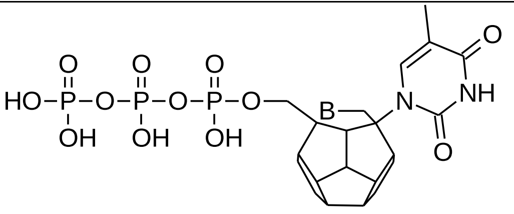
85	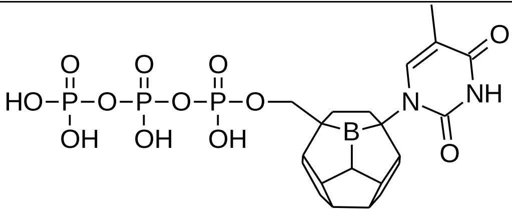	92	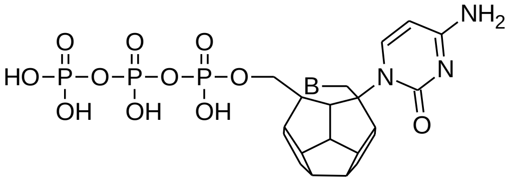
86	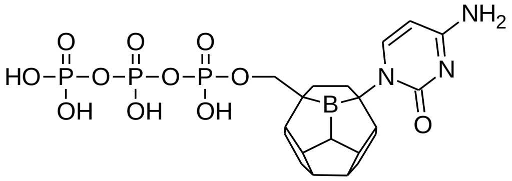	93	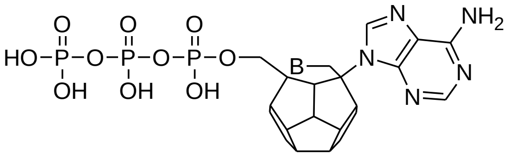
87	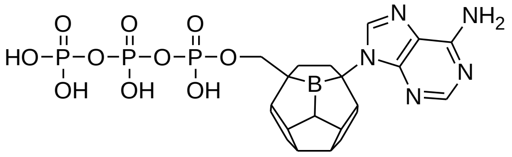	94	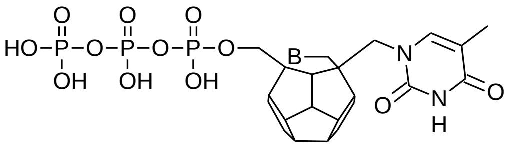
88	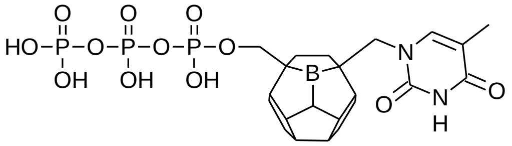	95	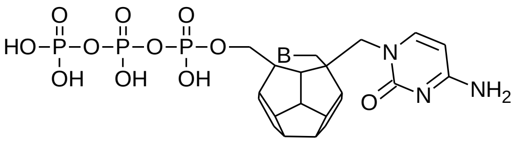
89	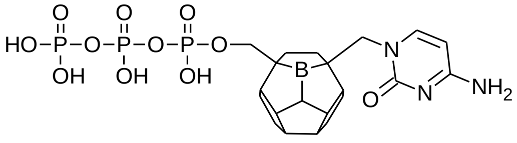	96	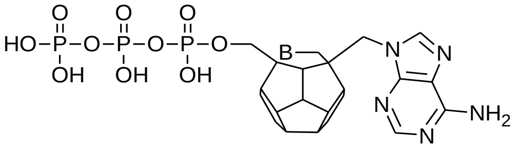
90	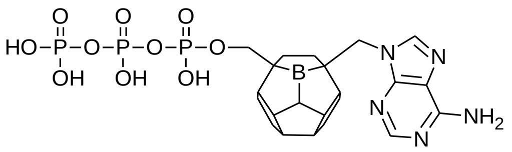	97	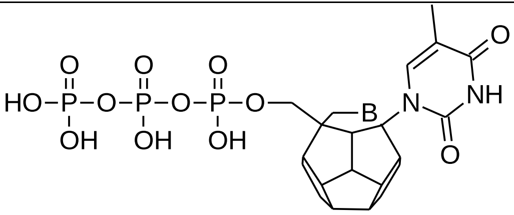

98	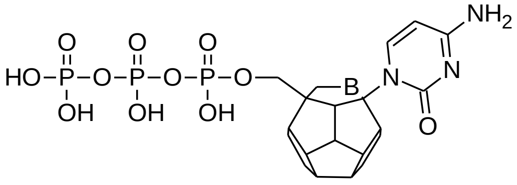
99	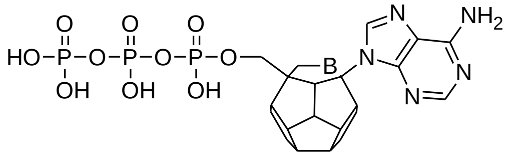
100	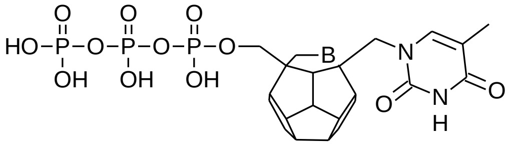
101	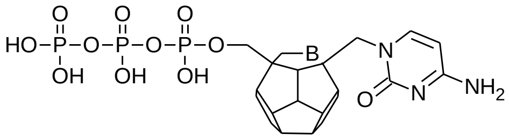
102	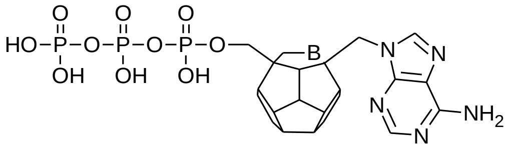
103	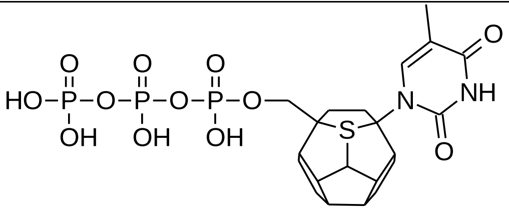
104	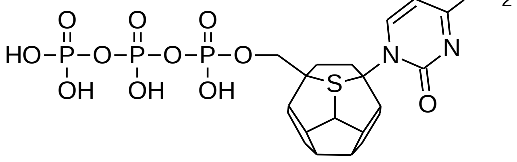
105	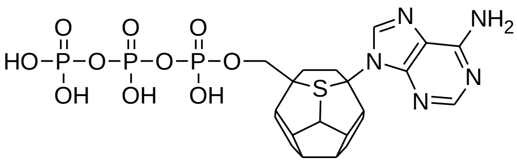
106	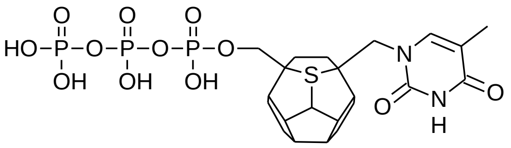
107	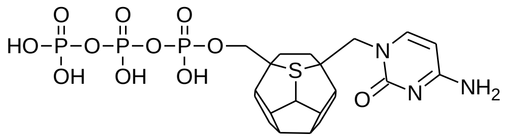
108	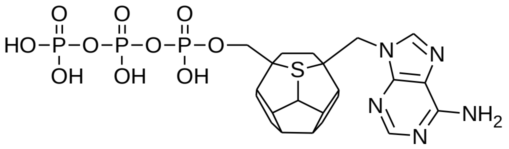
109	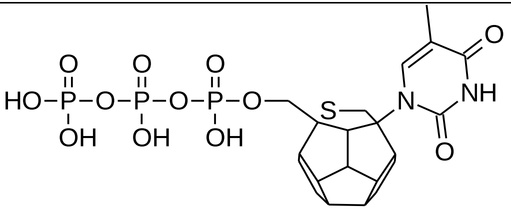
110	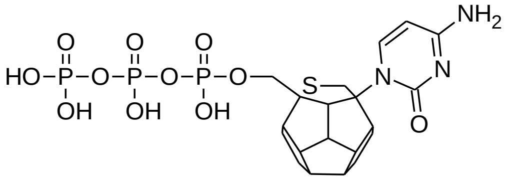
111	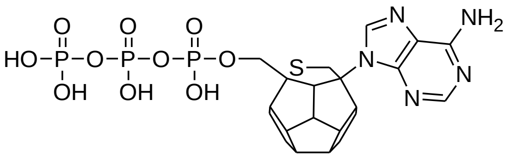

112	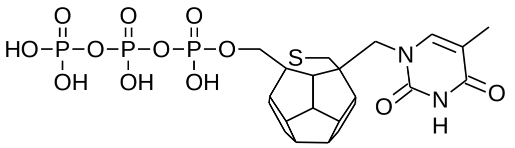
113	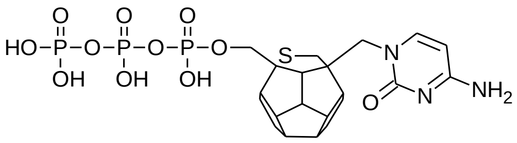
114	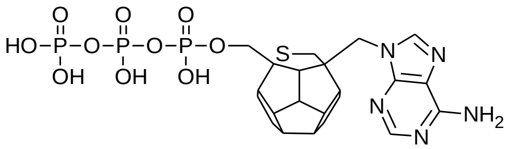
115	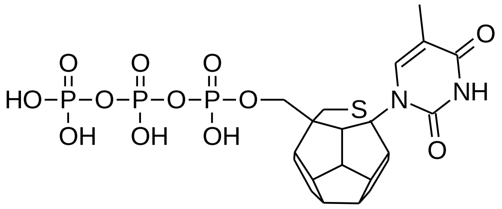
116	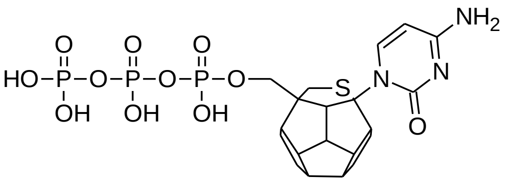
117	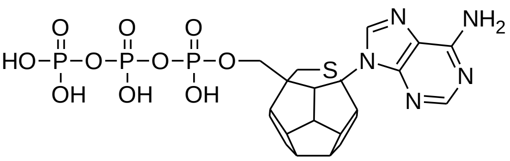
118	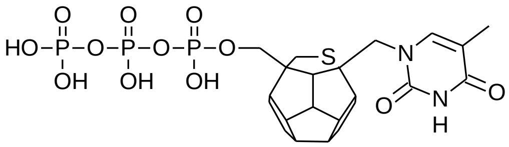

119	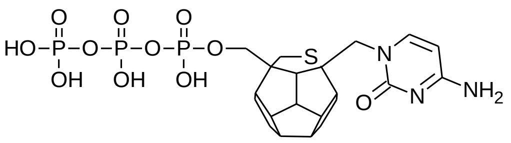
120	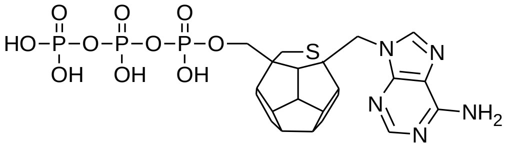
121	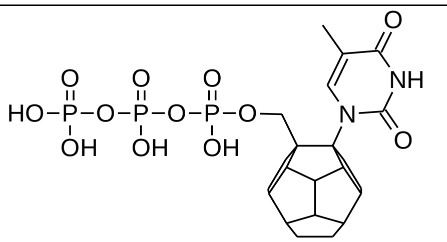
122	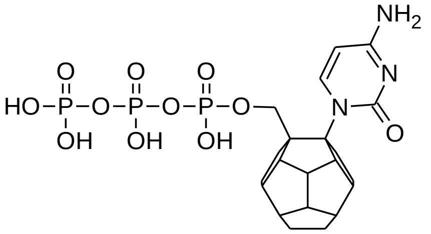
123	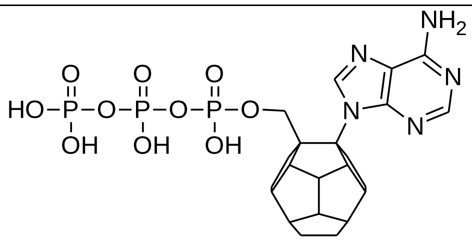
124	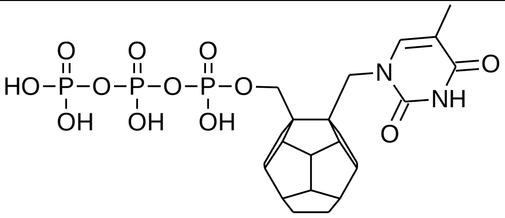
125	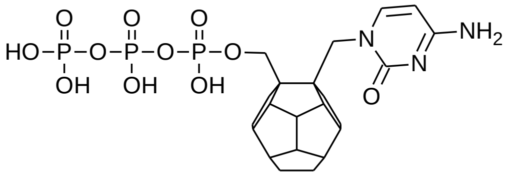

126	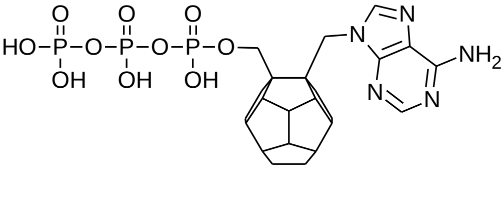
127	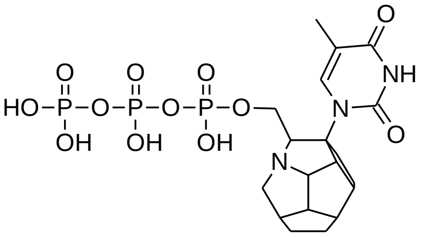
128	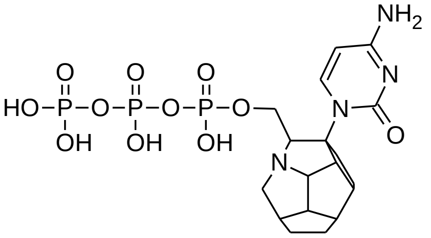
129	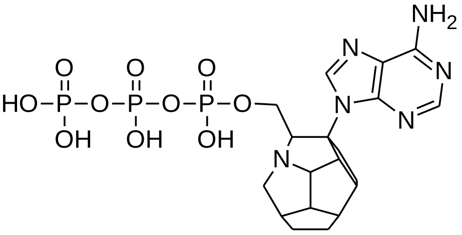
130	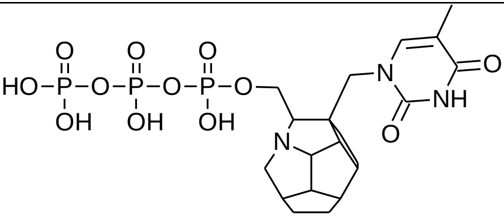
131	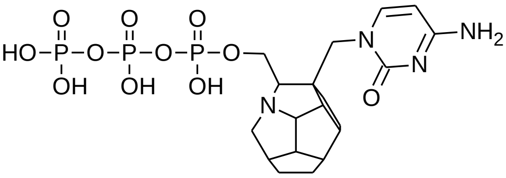
132	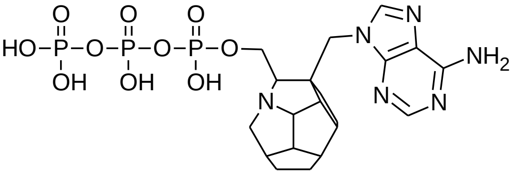

133	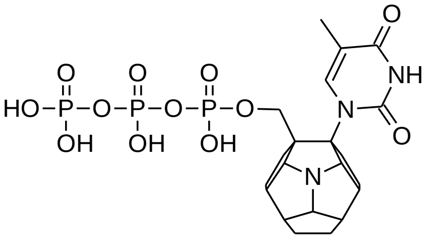
134	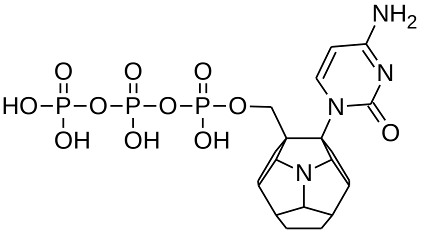
135	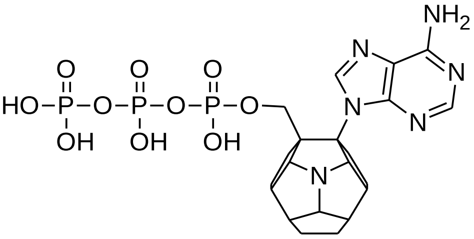
136	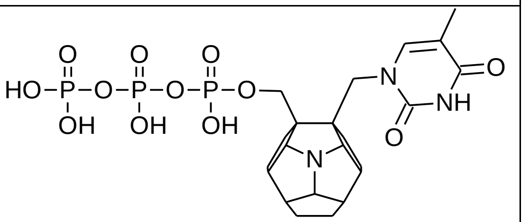
137	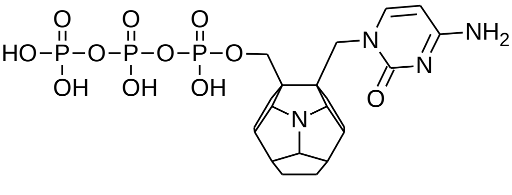
138	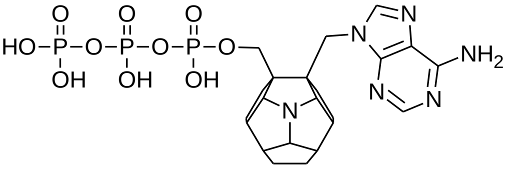
139	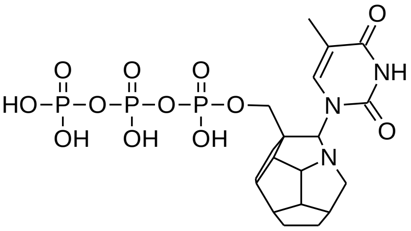

140	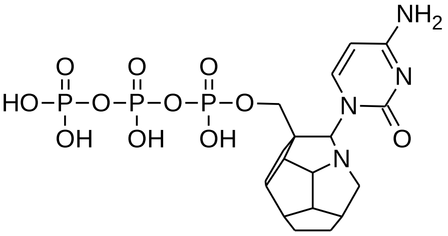
141	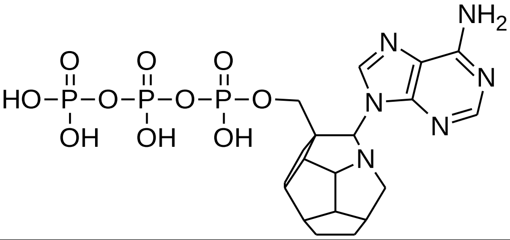
142	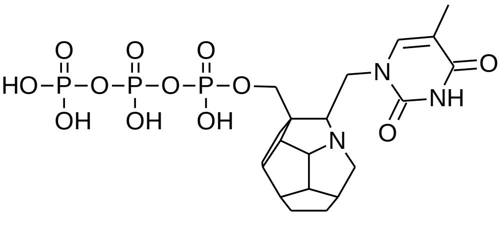
143	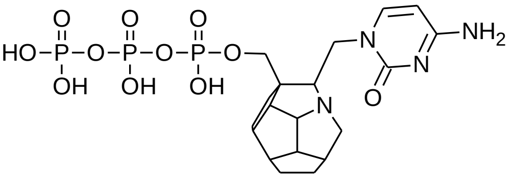
144	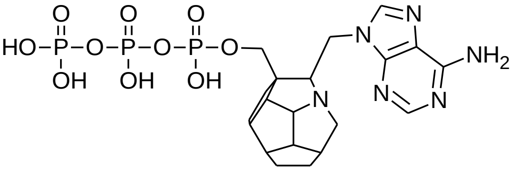
145	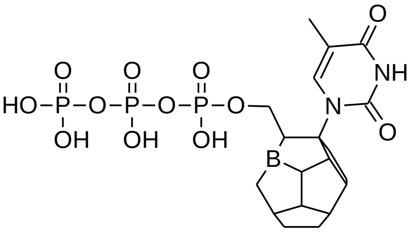
146	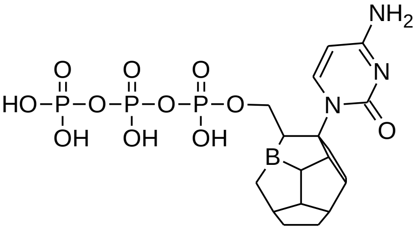
147	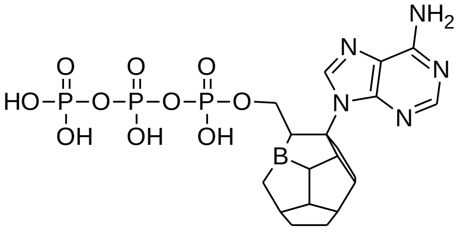
148	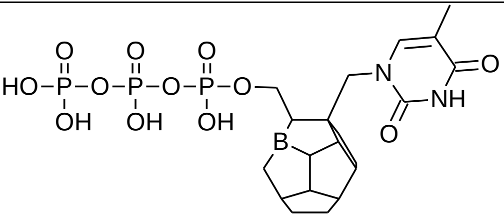
149	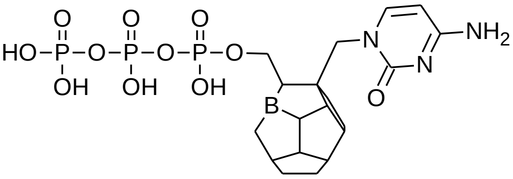
150	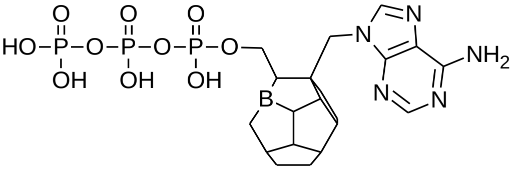
151	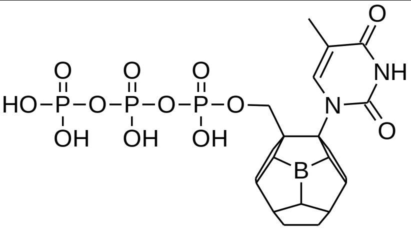
152	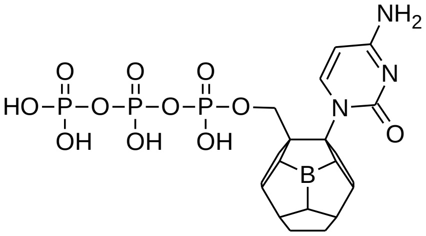
153	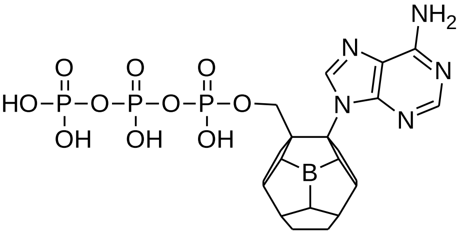

154	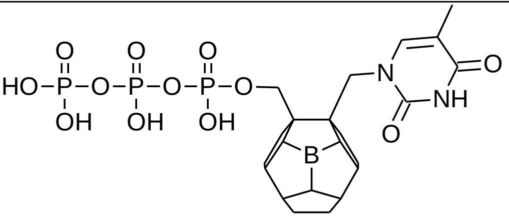
155	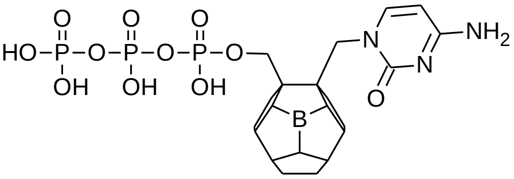
156	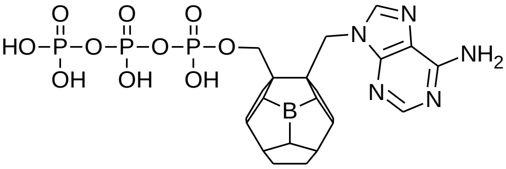
157	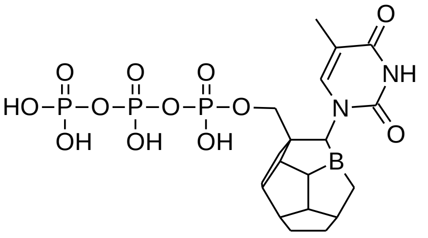
158	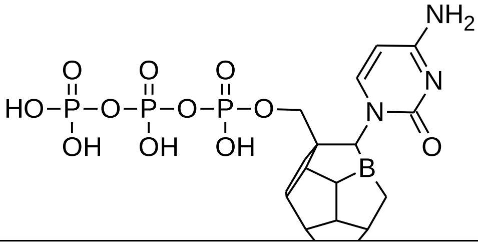
159	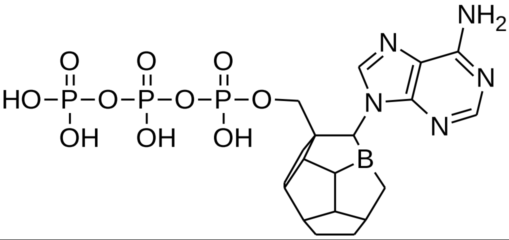
160	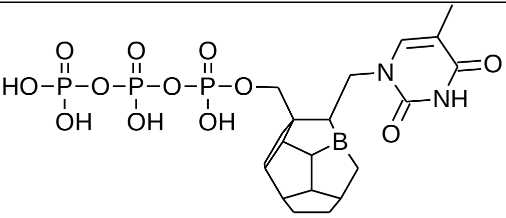

161	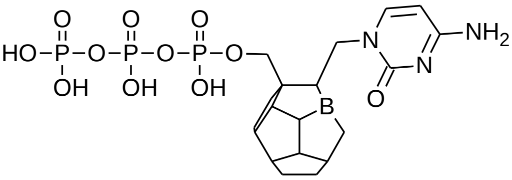
162	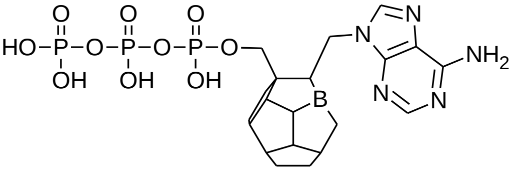
163	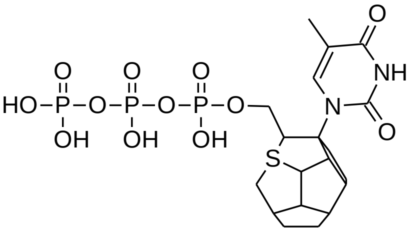
164	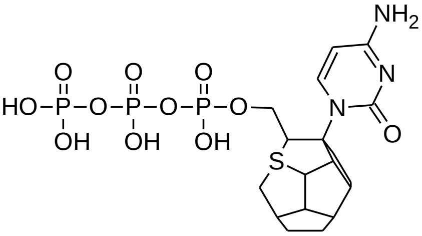
165	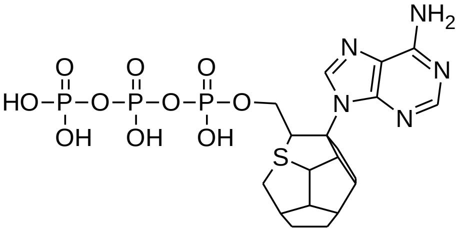
166	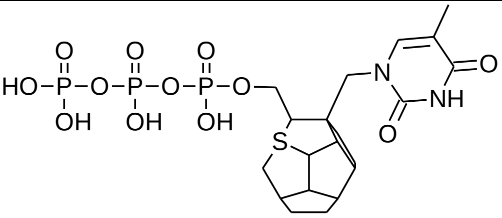
167	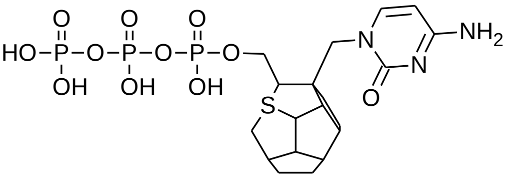

168	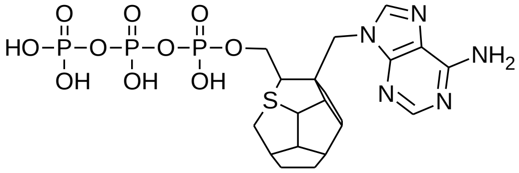
169	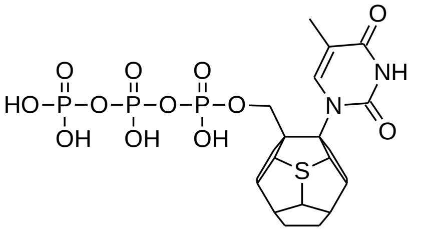
170	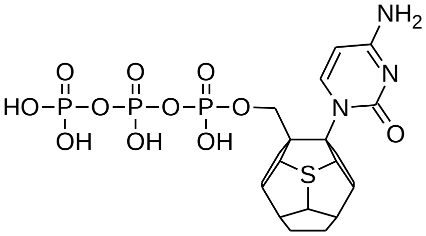
171	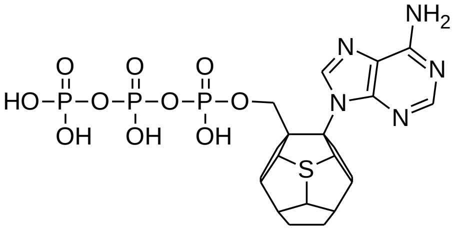
172	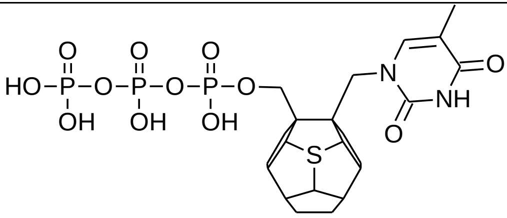
173	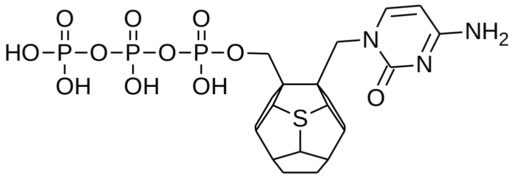
174	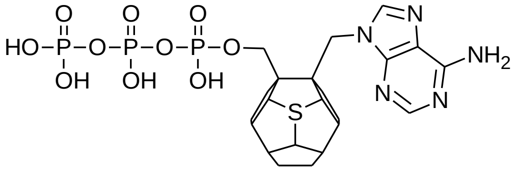
175	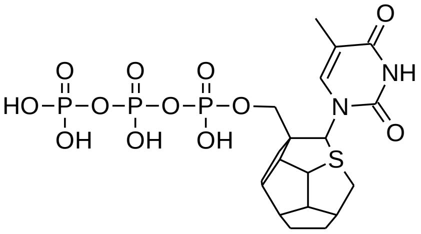
176	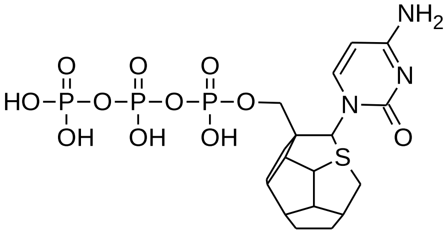
177	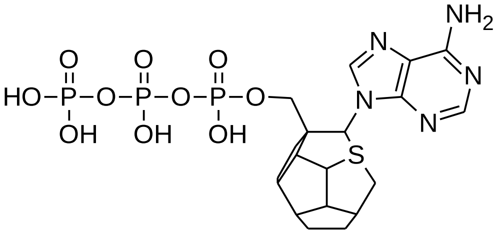
178	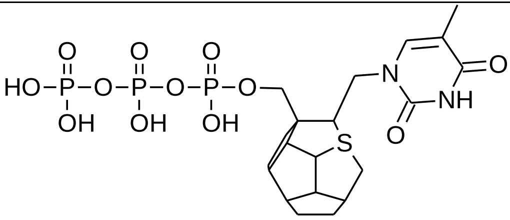
179	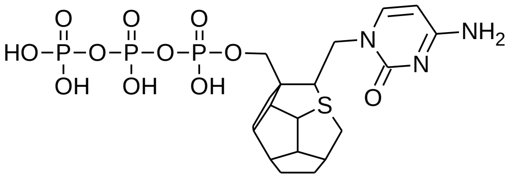
180	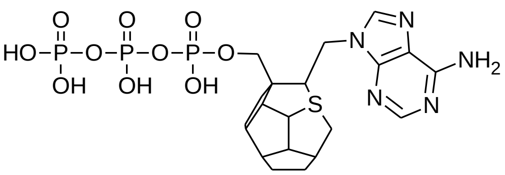

Table S2: Test ligands. dTTP - 2'-deoxythymidine triphosphate, ddATP - 2',3'-dideoxyadenosine triphosphate, ddUTP - 2',3'-dideoxyuridine triphosphate.

Name/ PubChem CID	Structure
11191118	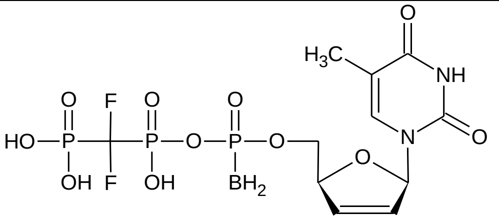
162005	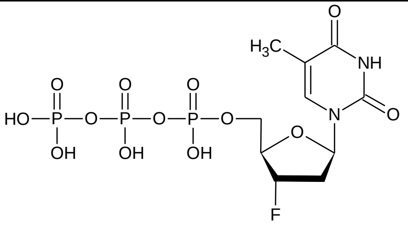
44400853	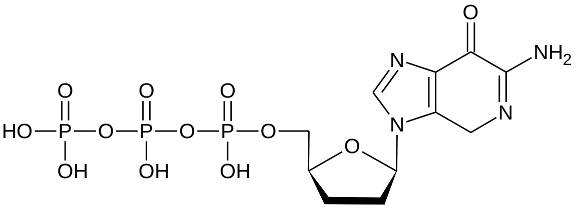
462076	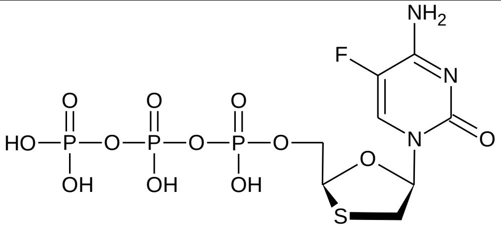
6338298	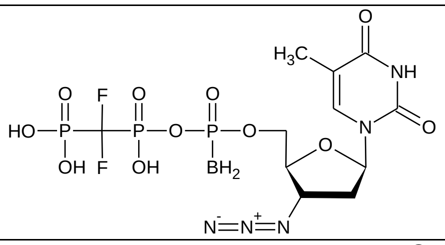
73345321	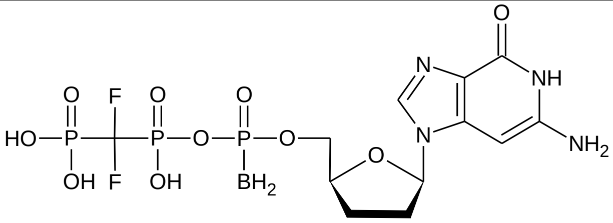
73346842	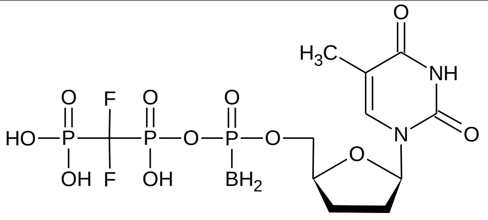

73348353	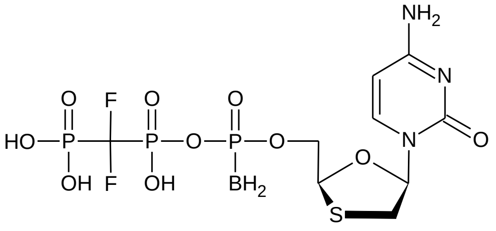
73349929	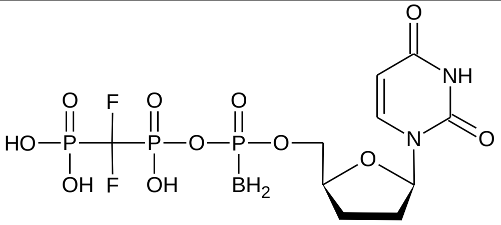
73352670	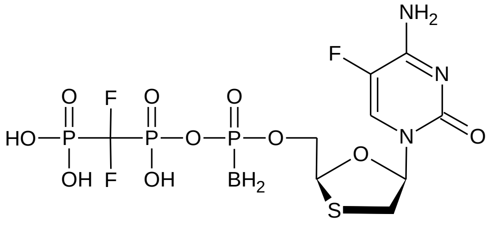
73352885	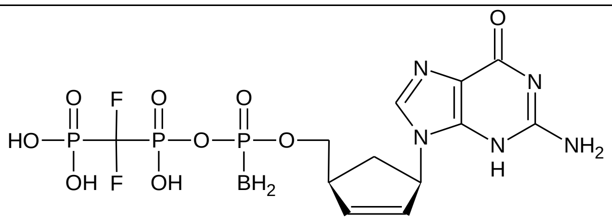
73352886	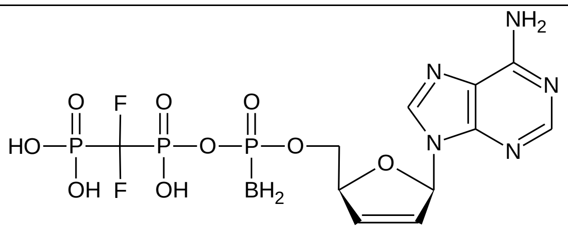
73354395	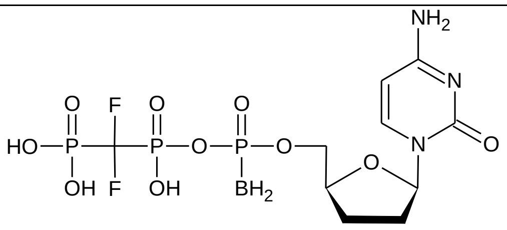
73354396	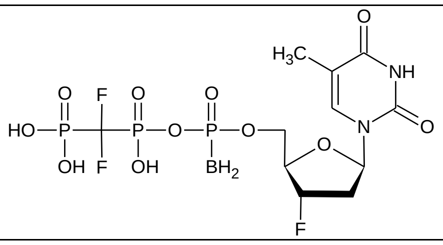

Lamivudine triphosphate	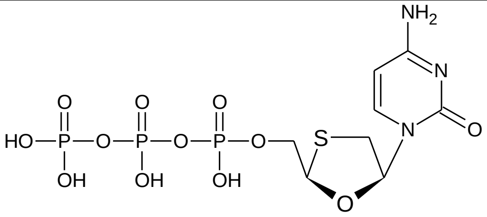
Stavudine triphosphate	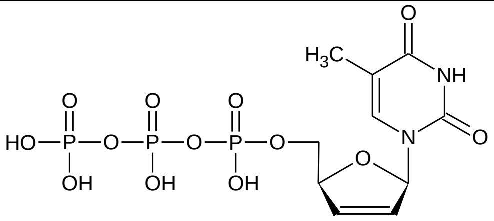
Zidovudine triphosphate	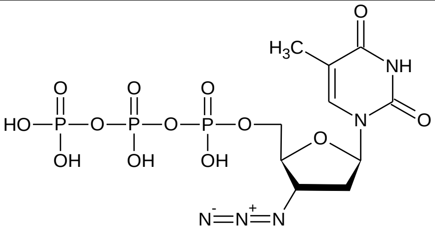
dTTP	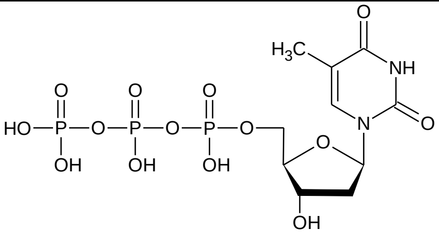
ddATP	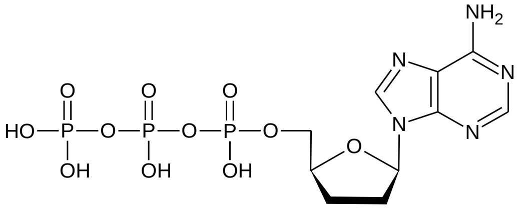
ddUTP	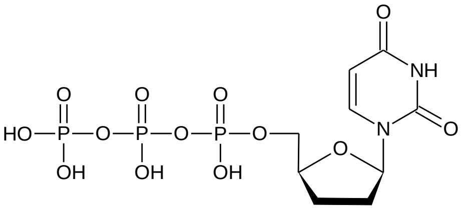

Table S3: Scoring function values for test compounds docked to 3KK1.

	Ki [nM]	LigScore1	LigScore2	-PLP1	-PLP2	Jain	-PMF	-PMF04
**11191118**	45	7,85	7,72	129,32	122,63	4,96	152,54	102,61
**162005**	71	7,84	7,94	142,22	129,14	9,23	173,82	126,01
**44400853**	25	7,80	8,34	152,28	127,40	7,96	166,46	119,45
**462076**	501	7,43	7,10	130,81	119,37	5,37	147,43	118,73
**6338298**	94	7,89	7,73	153,85	140,46	6,06	163,21	121,79
**73345321**	71	7,86	8,19	150,01	142,60	6,79	143,94	108,18
**73346842**	236	7,57	7,56	143,67	133,62	6,89	146,82	107,99
**73348353**	314	7,98	7,56	139,93	131,41	4,74	150,75	109,42
**73349929**	438	7,68	7,46	144,85	135,02	6,94	152,48	107,28
**73352670**	1770	7,55	7,42	129,13	111,07	6,46	138,05	113,04
**73352885**	34	7,82	7,71	140,13	139,24	6,66	144,72	101,84
**73352886**	47	7,69	7,89	135,08	125,47	5,45	148,49	109,55
**73354395**	23	7,40	7,44	125,58	123,40	6,59	148,20	102,65
**73354396**	46	7,67	7,55	117,35	110,65	7,24	167,94	120,36
**Lamivudine TP**	188	7,51	7,22	131,02	111,74	6,29	162,77	112,55
**Stavudine TP**	56	7,82	8,14	138,24	118,63	8,96	182,21	108,38
**Zidovudine TP**	91	7,63	7,69	151,06	138,80	6,90	193,02	133,90
**dTTP**	61	7,68	8,27	146,92	126,30	7,29	170,71	121,02
**ddATP**	20	7,82	8,52	147,58	122,25	7,71	168,49	125,30
**ddUTP**	545	7,78	8,15	129,12	121,09	6,50	163,65	114,23
**Pearson correlation coefficient**	-	-0,30026	-0,35891	-0,28044	-0,36593	-0,19670	**-0,41288**	-0,05902

Table S4: Scoring function values for test compounds docked to 3KK2.

	Ki [nM]	LigScore1	LigScore2	-PLP1	-PLP2	Jain	-PMF	-PMF04
**11191118**	45	7,89	7,93	136,76	115,69	4,81	147,93	99,52
**162005**	71	8,04	8,41	140,33	123,58	8,64	157,41	110,09
**44400853**	25	8,00	8,21	149,03	134,44	6,88	159,80	108,75
**462076**	501	7,62	7,51	135,57	123,30	5,29	146,64	111,09
**6338298**	94	7,86	7,37	148,61	135,30	8,98	148,12	105,48
**73345321**	71	8,03	7,86	143,06	122,96	7,31	144,77	103,58
**73346842**	236	7,83	8,30	143,62	119,22	7,81	147,23	94,23
**73348353**	314	7,51	7,66	130,18	110,14	3,70	137,43	100,62
**73349929**	438	7,83	7,80	124,90	112,92	5,61	146,26	100,45
**73352670**	1770	7,75	7,79	131,35	129,92	6,43	123,66	103,78
**73352885**	34	7,75	7,93	148,17	132,69	7,35	136,43	98,78
**73352886**	47	7,97	7,98	131,89	114,84	7,46	148,61	112,21
**73354395**	23	7,68	7,44	122,06	114,95	7,95	142,37	99,10
**73354396**	46	8,00	7,63	136,99	124,61	6,39	152,55	111,03
**Lamivudine TP**	188	7,68	7,41	127,92	111,95	4,43	149,13	105,63
**Stavudine TP**	56	8,00	8,09	137,08	120,85	5,32	168,54	97,44
**Zidovudine TP**	91	7,59	7,37	137,64	128,77	7,45	151,59	113,34
**dTTP**	61	8,01	8,33	140,39	129,35	6,74	174,72	108,77
**ddATP**	20	7,87	8,02	137,12	113,83	6,56	160,93	116,77
**ddUTP**	545	7,69	8,17	124,28	108,88	4,51	156,16	101,31
**Pearson correlation coefficient**	-	-0,32680	-0,08294	-0,33107	0,03659	-0,23164	**-0,57143**	-0,13186

Table S5: Scoring function values for test compounds docked to 3KJV.

	Ki [nM]	LigScore1	LigScore2	-PLP1	-PLP2	Jain	-PMF	-PMF04
**11191118**	45	6,86	6,74	93,58	83,28	2,51	138,85	91,38
**162005**	71	6,87	6,81	93,15	80,29	3,73	144,85	102,66
**44400853**	25	6,95	7,17	101,88	88,12	2,99	149,88	104,44
**462076**	501	6,93	6,66	77,80	77,02	2,72	132,11	107,92
**6338298**	94	6,89	6,67	103,84	91,28	4,31	112,05	89,49
**73345321**	71	7,07	7,06	94,18	90,71	4,85	131,36	103,36
**73346842**	236	7,11	6,89	89,38	84,05	2,38	129,74	82,40
**73348353**	314	7,02	6,80	96,07	82,98	3,66	119,14	94,11
**73349929**	438	6,97	6,66	88,12	80,53	5,88	133,78	91,27
**73352670**	1770	7,18	6,76	92,75	81,78	2,86	115,63	93,71
**73352885**	34	7,16	6,86	86,47	77,69	4,25	130,21	95,55
**73352886**	47	7,00	7,18	92,56	79,42	2,42	123,92	94,39
**73354395**	23	6,85	6,57	84,02	72,02	0,93	128,50	85,87
**73354396**	46	6,93	6,49	87,16	78,29	3,95	133,86	97,86
**Lamivudine TP**	188	6,69	6,36	86,18	75,79	1,91	129,64	92,82
**Stavudine TP**	56	6,96	6,62	85,11	80,41	5,00	138,65	89,72
**Zidovudine TP**	91	7,14	6,73	94,37	85,82	4,34	147,45	110,73
**dTTP**	61	7,20	6,73	79,59	81,50	6,04	141,66	95,09
**ddATP**	20	6,47	6,25	74,89	77,39	1,14	130,48	99,10
**ddUTP**	545	6,60	6,26	71,32	65,87	3,86	112,02	82,27
**Pearson correlation coefficient**	-	0,19670	-0,08830	-0,05609	-0,12437	-0,03737	**-0,47141**	-0,12190

Table S6: Scoring function values for test compounds docked to 1RTD.

	Ki [nM]	LigScore1	LigScore2	-PLP1	-PLP2	Jain	-PMF	-PMF04
**11191118**	45	7,07	7,09	135,64	116,82	7,94	137,65	116,46
**162005**	71	7,38	7,01	126,45	102,07	5,02	144,45	133,74
**44400853**	25	6,89	7,01	118,16	105,28	6,32	131,73	118,68
**462076**	501	6,89	6,75	115,93	100,65	3,68	128,32	119,89
**6338298**	94	7,79	7,35	123,70	107,6	4,60	154,75	137,30
**73345321**	71	7,27	7,08	111,70	97,08	4,88	143,05	136,88
**73346842**	236	7,40	7,24	131,90	108,32	5,75	136,99	125,55
**73348353**	314	7,29	6,63	117,89	101,49	4,91	125,01	122,27
**73349929**	438	7,12	6,67	106,38	97,02	5,15	139,79	120,35
**73352670**	1770	7,14	6,92	113,79	107,50	4,90	131,34	127,88
**73352885**	34	6,82	6,59	114,29	100,49	3,48	139,71	119,62
**73352886**	47	6,92	7,03	119,87	114,77	5,24	150,70	136,52
**73354395**	23	7,10	6,80	109,10	93,52	3,08	144,88	123,53
**73354396**	46	7,29	6,82	129,90	112,41	6,64	147,00	135,57
**Lamivudine TP**	188	7,09	6,64	109,09	99,18	3,45	139,47	126,22
**Stavudine TP**	56	7,32	7,11	123,66	103,35	4,61	142,83	115,62
**Zidovudine TP**	91	7,21	6,70	119,79	98,78	4,30	148,41	137,78
**dTTP**	61	7,06	6,80	115,91	105,00	3,59	149,37	129,21
**ddATP**	20	7,03	7,28	116,74	93,28	3,24	148,53	141,34
**ddUTP**	545	7,07	7,17	116,74	97,29	5,06	143,00	124,11
**Pearson correlation coefficient**	-	-0,04898	-0,08415	-0,24560	0,03089	-0,00056	**-0,46013**	-0,12617

Table S7: Scoring function values for test compounds docked to 1TO5.

	Ki [nM]	LigScore1	LigScore2	-PLP1	-PLP2	Jain	-PMF	-PMF04
**11191118**	45	7,59	7,54	135,61	119,78	6,20	167,88	100,73
**162005**	71	7,49	7,59	135,74	117,48	5,69	143,12	91,77
**44400853**	25	7,81	7,95	138,92	117,43	6,32	156,95	100,60
**462076**	501	7,19	7,13	117,64	100,94	4,76	149,39	102,55
**6338298**	94	7,6	7,40	142,91	135,88	6,61	151,40	114,28
**73345321**	71	7,47	7,22	140,22	120,30	6,21	153,42	105,21
**73346842**	236	7,77	7,48	133,04	125,42	6,59	146,32	95,85
**73348353**	314	7,11	7,43	110,64	103,69	7,28	157,22	113,32
**73349929**	438	7,31	7,59	124,34	122,68	6,32	175,34	107,05
**73352670**	1770	7,23	7,16	132,03	115,81	5,26	142,15	104,67
**73352885**	34	7,40	7,47	136,96	131,10	6,14	157,20	106,94
**73352886**	47	7,64	7,89	140,18	117,44	6,73	173,18	113,99
**73354395**	23	7,36	7,04	124,05	112,53	4,28	145,18	94,82
**73354396**	46	7,84	7,36	141,17	119,35	6,19	169,43	110,54
**Lamivudine TP**	188	7,37	7,27	109,02	105,34	6,93	167,25	105,30
**Stavudine TP**	56	7,48	7,39	121,47	111,52	6,39	162,72	98,05
**Zidovudine TP**	91	7,68	7,42	144,49	119,58	7,84	147,64	99,09
**dTTP**	61	7,67	7,57	134,92	111,89	5,51	152,02	99,23
**ddATP**	20	7,37	7,46	130,90	115,16	5,68	147,08	103,41
**ddUTP**	545	7,64	7,86	132,01	109,54	7,13	132,14	90,68
**Pearson correlation coefficient**	-	**-0,42560**	-0,24884	-0,18413	-0,18792	-0,18450	-0,31409	0,00830

Table S8: Scoring function values for test compounds docked to 3V4I.

	Ki [nM]	LigScore1	LigScore2	-PLP1	-PLP2	Jain	-PMF	-PMF04
**11191118**	45	7,60	7,44	127,35	107,58	5,34	159,50	132,65
**162005**	71	7,35	7,54	133,61	116,93	4,63	171,02	153,36
**44400853**	25	7,11	6,75	121,63	107,37	6,55	173,18	133,81
**462076**	501	7,41	7,18	117,70	113,04	6,00	162,65	138,79
**6338298**	94	6,93	6,56	132,17	115,00	5,05	161,88	146,65
**73345321**	71	7,43	7,21	131,06	115,12	4,75	159,12	141,47
**73346842**	236	7,09	7,00	114,28	116,89	6,63	143,14	117,07
**73348353**	314	7,31	6,78	107,79	109,41	4,54	143,21	129,11
**73349929**	438	7,57	7,57	121,11	110,60	7,34	152,23	131,46
**73352670**	1770	6,95	7,06	110,64	107,55	6,30	150,93	150,55
**73352885**	34	7,48	7,15	125,91	129,32	4,35	132,33	117,95
**73352886**	47	6,60	6,98	129,14	114,28	6,42	170,49	154,97
**73354395**	23	7,05	6,96	128,09	110,49	6,30	164,15	132,46
**73354396**	46	7,53	7,09	135,12	117,38	5,01	150,99	144,90
**Lamivudine TP**	188	7,32	7,19	118,64	108,61	5,53	161,72	127,54
**Stavudine TP**	56	7,17	7,02	119,00	95,48	6,15	158,31	118,28
**Zidovudine TP**	91	7,38	7,24	134,65	126,12	5,57	184,87	156,65
**dTTP**	61	6,92	6,57	114,36	106,78	6,20	184,20	141,72
**ddATP**	20	7,56	7,28	124,37	97,66	8,23	163,99	145,54
**ddUTP**	545	7,34	7,66	118,71	104,13	3,23	170,33	135,83
**Pearson correlation coefficient**	-	-0,15255	0,12387	**-0,53376**	-0,14607	0,02828	-0,21577	0,15436

Table S9: Scoring function values for test compounds docked to Model 1.

	Ki [nM]	LigScore1	LigScore2	-PLP1	-PLP2	Jain	-PMF	-PMF04
**11191118**	45	7,05	5,98	110,76	105,53	6,78	126,05	79,26
**162005**	71	7,10	6,03	98,58	99,56	6,31	127,54	81,14
**44400853**	25	6,97	5,73	126,29	110,26	5,80	129,30	86,33
**462076**	501	7,25	6,43	103,07	100,71	6,35	121,81	94,92
**6338298**	94	7,32	6,02	125,78	103,14	5,18	130,23	94,15
**73345321**	71	6,95	6,15	104,31	99,05	7,80	118,35	71,61
**73346842**	236	7,56	6,70	117,59	101,25	5,80	129,47	86,23
**73348353**	314	7,46	6,45	114,38	103,28	3,88	114,51	84,50
**73349929**	438	7,18	6,18	113,25	107,6	5,16	123,54	81,26
**73352670**	1770	7,49	6,48	106,41	106,16	5,12	112,86	93,30
**73352885**	34	7,25	6,47	121,78	116,59	6,12	113,49	78,93
**73352886**	47	7,45	6,97	110,95	105,81	4,33	121,74	84,46
**73354395**	23	7,58	6,95	111,21	98,53	6,03	123,19	79,12
**73354396**	46	7,30	6,83	122,01	107,46	6,80	129,28	96,65
**Lamivudine TP**	188	7,02	6,81	100,47	98,38	3,16	120,95	78,41
**Stavudine TP**	56	6,86	6,54	110,31	101,61	6,86	137,90	78,19
**Zidovudine TP**	91	6,89	5,50	117,32	103,78	7,02	132,74	88,99
**dTTP**	61	6,99	6,30	121,15	104,78	5,44	144,93	90,33
**ddATP**	20	6,91	5,98	111,56	92,70	4,50	124,12	86,76
**ddUTP**	545	7,34	6,34	103,48	96,41	4,15	125,94	74,65
**Pearson correlation coefficient**	-	0,38784	0,10549	-0,30872	0,03603	-0,23478	**-0,42549**	0,25771

Table S10: Scoring function values for test compounds docked to Model 2.

	Ki [nM]	LigScore1	LigScore2	-PLP1	-PLP2	Jain	-PMF	-PMF04
**11191118**	45	7,83	7,61	131,35	114,69	5,46	128,34	89,24
**162005**	71	7,69	7,88	146,38	130,43	6,91	162,67	111,24
**44400853**	25	7,88	8,02	148,64	134,21	6,14	149,65	101,75
**462076**	501	7,06	7,81	140,45	124,84	4,09	138,32	110,92
**6338298**	94	7,29	7,94	134,46	122,33	6,50	148,81	106,91
**73345321**	71	8,00	8,15	143,73	134,21	7,14	160,18	115,12
**73346842**	236	7,52	7,74	124,38	110,62	6,29	153,34	94,23
**73348353**	314	7,84	7,33	125,11	108,74	4,24	143,60	94,26
**73349929**	438	7,19	7,35	121,86	113,53	5,86	149,50	107,42
**73352670**	1770	7,45	7,79	126,68	115,65	5,40	118,35	96,82
**73352885**	34	7,55	7,40	149,44	136,05	6,56	147,56	104,54
**73352886**	47	7,96	7,90	131,19	113,37	4,50	147,51	106,63
**73354395**	23	7,54	7,49	127,76	110,18	5,54	138,11	85,36
**73354396**	46	7,83	7,55	127,45	123,81	5,47	148,64	100,55
**Lamivudine TP**	188	7,38	7,05	136,54	118,46	7,11	149,02	101,30
**Stavudine TP**	56	7,85	7,81	137,67	126,36	4,69	168,59	101,04
**Zidovudine TP**	91	7,95	8,04	152,40	138,85	4,41	183,68	117,68
**dTTP**	61	7,52	8,07	148,86	131,26	6,52	172,78	116,81
**ddATP**	20	7,58	8,06	141,55	127,08	5,99	167,71	122,20
**ddUTP**	545	7,40	7,89	129,97	115,60	7,26	154,75	100,16
**Pearson correlation coefficient**	-	-0,38585	-0,06393	-0,38193	-0,32080	-0,11134	**-0,54346**	-0,19145

Table S11: Scoring function values for test compounds docked to Model 3.

	Ki [nM]	LigScore1	LigScore2	-PLP1	-PLP2	Jain	-PMF	-PMF04
**11191118**	45	7,25	7,18	110,15	108,27	4,25	109,37	70,91
**162005**	71	7,58	7,57	110,94	99,90	3,55	129,23	84,64
**44400853**	25	7,62	7,71	118,73	105,91	4,11	119,09	80,24
**462076**	501	7,56	7,32	111,18	98,17	3,76	106,47	77,48
**6338298**	94	7,61	7,98	131,87	119,17	4,35	116,87	87,79
**73345321**	71	7,64	7,65	112,14	94,75	3,58	115,75	71,01
**73346842**	236	7,14	7,45	111,79	100,01	4,30	118,82	76,74
**73348353**	314	7,55	7,37	124,01	112,08	4,06	113,25	71,64
**73349929**	438	7,16	7,14	110,65	109,86	4,24	115,24	66,25
**73352670**	1770	7,63	7,50	111,83	104,93	4,67	98,43	75,47
**73352885**	34	7,32	7,34	131,91	116,67	3,50	116,74	76,30
**73352886**	47	7,28	7,76	115,96	104,56	4,60	115,49	76,95
**73354395**	23	7,10	6,64	110,57	102,79	3,62	113,28	65,72
**73354396**	46	7,16	7,14	110,70	101,92	4,26	120,09	85,01
**Lamivudine TP**	188	7,34	7,06	109,80	94,49	3,70	112,59	71,69
**Stavudine TP**	56	7,15	7,13	112,20	95,51	5,10	130,81	71,54
**Zidovudine TP**	91	7,40	7,25	117,79	102,99	2,71	119,66	71,33
**dTTP**	61	7,71	7,46	127,18	105,76	5,90	125,59	84,54
**ddATP**	20	7,3	7,28	112,18	99,93	5,19	122,01	85,38
**ddUTP**	545	7,26	7,22	101,63	93,48	4,34	117,39	73,36
**Pearson correlation coefficient**	-	0,23445	0,04568	-0,23945	-0,03818	0,11000	**-0,66910**	-0,14801

Table S12: Scoring function values for test compounds docked to Model 4.

	Ki [nM]	LigScore1	LigScore2	-PLP1	-PLP2	Jain	-PMF	-PMF04
**11191118**	45	7,38	7,52	129,39	110,62	4,80	127,80	78,68
**162005**	71	7,47	7,48	135,13	124,37	6,18	147,98	101,66
**44400853**	25	7,87	7,82	148,77	137,85	7,00	145,59	96,13
**462076**	501	7,54	7,68	131,92	122,00	5,45	136,96	99,77
**6338298**	94	7,73	7,55	142,84	126,27	5,00	140,47	95,85
**73345321**	71	7,62	7,46	147,34	134,55	7,55	112,88	83,10
**73346842**	236	7,41	7,10	132,57	123,93	4,24	142,01	90,85
**73348353**	314	7,60	7,38	135,87	126,05	6,12	147,68	96,50
**73349929**	438	7,34	8,36	144,19	136,10	6,23	126,90	86,36
**73352670**	1770	7,51	7,59	133,70	121,71	5,28	135,56	98,42
**73352885**	34	7,86	7,92	145,98	129,50	7,47	131,36	86,52
**73352886**	47	8,10	7,96	147,02	130,43	7,90	152,92	105,43
**73354395**	23	7,36	7,56	133,53	123,00	6,97	157,08	95,69
**73354396**	46	7,70	7,42	124,63	117,35	3,74	135,55	94,23
**Lamivudine TP**	188	7,51	7,52	130,91	114,39	5,59	135,42	93,01
**Stavudine TP**	56	7,22	7,75	142,40	133,19	6,36	151,74	88,70
**Zidovudine TP**	91	7,39	7,38	137,54	109,97	6,86	162,63	112,63
**dTTP**	61	7,38	7,21	132,92	120,24	7,50	168,41	103,28
**ddATP**	20	7,50	8,03	149,29	130,82	8,10	142,61	99,19
**ddUTP**	545	7,40	8,17	133,88	117,27	4,82	149,05	92,87
**Pearson correlation coefficient**	-	-0,14516	0,09644	-0,22613	-0,11149	**-0,31380**	-0,16931	0,06468

Table S13: Results of docking to 3KK2.

	ID	-PMF	ID	-PMF	ID	-PMF
	1	159,81	61	159,96	121	179,48
	2	194,64	62	159,46	122	184,04
	3	165,14	63	176,24	123	177,91
	4	189,20	64	174,54	124	175,87
	5	185,71	65	183,95	125	181,46
	6	197,88	66	176,13	126	187,87
Nitrogen derivatives	7	190,06	67	165,90	127	184,12
	8	164,42	68	168,77	128	192,85
	9	182,59	69	181,79	129	179,86
	10	163,38	70	188,70	130	187,30
	11	175,67	71	164,67	131	179,12
	12	126,45	72	168,74	132	178,73
	13	153,88	73	186,51	133	176,01
	14	167,09	74	182,75	134	182,69
	15	191,45	75	168,80	135	190,28
	16	183,26	76	162,71	136	155,19
	17	159,71	77	170,27	137	194,62
	18	174,92	78	197,16	138	170,58
	19	150,28	79	166,16	139	182,27
	20	145,7	80	176,22	140	198,41
	21	169,46	81	172,24	141	173,89
	22	160,60	82	185,56	142	173,26
	23	191,08	83	189,50	143	172,44
	24	173,36	84	175,09	144	181,78
Boron derivatives	25	191,36	85	164,77	145	175,05
	26	181,75	86	167,62	146	185,19
	27	183,42	87	177,56	147	183,30
	28	190,09	88	170,78	148	159,37
	29	181,57	89	185,91	149	177,88
	30	171,06	90	174,03	150	175,20
	31	158,57	91	165,27	151	163,64
	32	165,92	92	173,28	152	147,74
	33	174,31	93	183,19	153	183,61
	34	162,37	94	184,85	154	159,14
	35	167,89	95	178,84	155	189,79
	36	182,84	96	167,58	156	176,59
	37	158,57	97	164,45	157	191,93
	38	175,38	98	189,20	158	166,28
	39	175,47	99	172,34	159	175,24
	40	182,55	100	183,42	160	179,72
	41	160,11	101	164,14	161	190,29
	42	191,39	102	190,89	162	175,10

Sulphur derivatives	43	144,79	103	181,79	163	175,66
	44	180,93	104	156,21	164	188,77
	45	179,62	105	175,86	165	188,01
	46	178,30	106	190,73	166	183,33
	47	186,98	107	189,51	167	165,17
	48	189,26	108	186,33	168	188,23
	49	193,88	109	166,27	169	174,29
	50	180,08	110	180,94	170	184,18
	51	192,12	111	174,86	171	187,01
	52	168,20	112	170,57	172	175,24
	53	179,58	113	183,88	173	194,10
	54	176,71	114	171,32	174	190,24
	55	189,67	115	169,49	175	191,84
	56	162,97	116	171,07	176	191,36
	57	192,87	117	167,30	177	185,43
	58	177,15	118	176,42	178	177,95
	59	183,14	119	165,47	179	196,89
	60	187,37	120	167,39	180	184,25

Table S14: Results of docking to Model 2.

	ID	-PMF	ID	-PMF	ID	-PMF
	1	163,97	61	179,36	121	174,16
	2	163,72	62	185,91	122	177,12
	3	179,73	63	180,56	123	181,48
	4	191,35	64	180,59	124	177,09
	5	199,49	65	189,21	125	179,04
	6	173,01	66	196,39	126	183,62
Nitrogen derivatives	7	179,02	67	162,95	127	138,55
	8	177,61	68	188,83	128	195,06
	9	172,18	69	165,47	129	180,73
	10	182,99	70	180,19	130	181,48
	11	181,34	71	192,59	131	185,90
	12	183,15	72	187,06	132	194,71
	13	164,67	73	184,24	133	191,28
	14	176,40	74	162,65	134	178,49
	15	188,35	75	170,05	135	176,36
	16	190,68	76	185,88	136	178,82
	17	184,14	77	186,14	137	199,25
	18	163,39	78	193,67	138	170,63

	19	160,22	79	170,02	139	171,89
	20	190,66	80	177,71	140	179,33
	21	168,16	81	191,18	141	169,85
	22	178,73	82	192,94	142	186,78
	23	182,28	83	198,07	143	181,42
	24	175,76	84	164,56	144	190,96
Boron derivatives	25	164,19	85	159,52	145	179,59
	26	163,96	86	161,96	146	178,95
	27	166,06	87	169,72	147	170,37
	28	186,96	88	167,04	148	173,75
	29	208,45	89	191,62	149	187,46
	30	191,76	90	180,90	150	190,31
	31	178,22	91	189,02	151	171,77
	32	179,31	92	166,26	152	187,57
	33	173,39	93	184,33	153	185,81
	34	173,90	94	191,20	154	175,07
	35	184,31	95	165,72	155	184,55
	36	174,05	96	205,23	156	186,57
	37	161,41	97	182,59	157	166,42
	38	167,98	98	168,94	158	161,17
	39	167,03	99	166,22	159	189,17
	40	172,92	100	190,17	160	179,02
	41	167,71	101	185,86	161	178,26
	42	180,11	102	188,32	162	174,11
Sulphur derivatives	43	174,36	103	167,74	163	170,29
	44	186,23	104	186,30	164	191,21
	45	181,77	105	161,23	165	175,29
	46	188,67	106	174,12	166	182,45
	47	203,10	107	163,77	167	173,07
	48	146,37	108	181,90	168	192,60
	49	193,66	109	168,03	169	181,62
	50	146,53	110	163,01	170	190,78
	51	153,09	111	173,01	171	181,84
	52	158,62	112	165,70	172	174,36
	53	172,93	113	166,61	173	181,45
	54	155,71	114	186,77	174	153,20
	55	178,46	115	183,04	175	169,12
	56	155,85	116	188,61	176	161,05
	57	185,24	117	171,37	177	191,98
	58	173,96	118	159,93	178	182,75
	59	187,06	119	179,51	179	184,60
	60	182,41	120	189,17	180	186,90

Table S15: Results of docking to Model 3.

	ID	-PMF	ID	-PMF	ID	-PMF
	1	158,80	61	157,03	121	153,92
	2	162,48	62	159,94	122	159,17
	3	165,96	63	152,13	123	159,85
	4	169,64	64	150,29	124	136,39
	5	164,67	65	154,55	125	178,34
	6	185,19	66	165,70	126	156,58
Nitrogen derivatives	7	140,52	67	158,12	127	149,40
	8	152,61	68	163,97	128	143,33
	9	166,13	69	161,66	129	143,62
	10	156,68	70	165,00	130	152,49
	11	159,01	71	162,82	131	149,15
	12	161,76	72	174,43	132	156,66
	13	170,04	73	171,93	133	160,00
	14	158,28	74	154,39	134	170,87
	15	159,01	75	167,97	135	149,32
	16	152,31	76	173,42	136	146,59
	17	162,04	77	166,76	137	139,25
	18	149,00	78	183,40	138	152,88
	19	156,35	79	164,73	139	151,31
	20	150,77	80	158,82	140	148,08
	21	165,81	81	164,85	141	154,24
	22	160,16	82	167,86	142	148,67
	23	141,34	83	172,67	143	159,31
	24	167,59	84	177,27	144	158,44
Boron derivatives	25	166,45	85	154,97	145	158,13
	26	144,15	86	154,50	146	134,72
	27	162,01	87	159,60	147	133,28
	28	163,93	88	165,48	148	152,55
	29	166,09	89	163,85	149	147,84
	30	171,78	90	172,33	150	173,51
	31	156,06	91	154,21	151	150,16
	32	146,64	92	165,51	152	168,56
	33	161,77	93	164,04	153	157,07
	34	149,56	94	168,90	154	143,46
	35	150,79	95	152,97	155	157,50
	36	153,99	96	173,28	156	161,71
	37	163,49	97	165,11	157	147,60
	38	154,35	98	159,02	158	152,93
	39	160,05	99	163,37	159	139,41
	40	151,04	100	166,22	160	147,72
	41	165,65	101	168,42	161	143,94
	42	167,32	102	179,27	162	157,30

Sulphur derivatives	43	163,86	103	170,83	163	148,06
	44	155,14	104	145,09	164	175,34
	45	159,58	105	173,08	165	153,88
	46	174,46	106	168,01	166	173,85
	47	162,20	107	173,80	167	154,81
	48	166,93	108	154,71	168	142,94
	49	164,78	109	156,51	169	157,43
	50	148,14	110	158,72	170	139,23
	51	158,30	111	156,76	171	156,25
	52	164,07	112	158,38	172	166,20
	53	155,94	113	163,85	173	148,52
	54	172,41	114	175,31	174	154,25
	55	163,34	115	160,71	175	151,64
	56	167,54	116	160,06	176	144,32
	57	158,47	117	160,99	177	143,50
	58	157,93	118	147,83	178	164,18
	59	158,69	119	163,47	179	155,72
	60	175,89	120	170,57	180	151,59

Table S16: Results of docking to 3V4I.

	ID	-PLP1	ID	-PLP1	ID	-PLP1
	1	116,61	61	110,75	121	83,72
	2	117,20	62	140,01	122	111,54
	3	130,55	63	133,18	123	98,48
	4	115,46	64	109,38	124	104,22
	5	116,01	65	127,56	125	113,96
	6	114,23	66	111,72	126	127,79
Nitrogen derivatives	7	139,14	67	127,00	127	111,54
	8	130,48	68	132,88	128	91,54
	9	136,82	69	117,77	129	134,50
	10	101,61	70	98,21	130	79,85
	11	129,78	71	118,55	131	114,95
	12	129,32	72	120,65	132	119,30
	13	126,15	73	106,18	133	108,70
	14	128,83	74	103,28	134	114,63
	15	138,59	75	90,82	135	114,54
	16	111,83	76	86,63	136	123,47
	17	121,18	77	100,14	137	105,36
	18	133,72	78	95,51	138	121,45
	19	126,80	79	102,51	139	106,01
	20	131,23	80	112,41	140	106,67

	21	135,06	81	98,77	141	124,63
	22	114,79	82	133,56	142	126,28
	23	112,49	83	78,95	143	109,44
	24	136,91	84	103,63	144	106,51
Boron derivatives	25	121,32	85	127,93	145	113,93
	26	119,77	86	123,62	146	91,89
	27	127,57	87	128,95	147	120,87
	28	107,75	88	119,40	148	114,24
	29	113,58	89	107,40	149	126,32
	30	109,27	90	128,19	150	124,86
	31	108,94	91	114,55	151	106,79
	32	97,19	92	116,50	152	117,28
	33	119,80	93	117,88	153	134,45
	34	105,88	94	97,54	154	118,72
	35	85,44	95	102,21	155	99,79
	36	118,23	96	104,58	156	131,10
	37	118,08	97	132,52	157	127,92
	38	121,36	98	123,38	158	97,92
	39	117,27	99	102,70	159	120,84
	40	118,30	100	106,16	160	93,07
	41	84,46	101	116,48	161	75,03
	42	101,80	102	114,29	162	116,10
Sulphur derivatives	43	125,47	103	108,13	163	110,01
	44	106,44	104	95,13	164	98,08
	45	107,61	105	112,46	165	119,18
	46	117,59	106	101,99	166	110,43
	47	92,20	107	85,00	167	73,13
	48	116,17	108	85,83	168	115,41
	49	109,85	109	96,81	169	106,29
	50	111,01	110	93,36	170	74,83
	51	108,31	111	120,13	171	112,07
	52	104,76	112	130,48	172	107,30
	53	101,40	113	81,85	173	127,30
	54	108,55	114	127,41	174	113,96
	55	123,66	115	124,67	175	108,88
	56	104,80	116	93,27	176	110,03
	57	102,95	117	102,59	177	131,87
	58	105,72	118	76,98	178	116,16
	59	107,68	119	90,28	179	109,56
	60	100,19	120	131,61	180	120,29
